# A reference floral transcriptome of sexual and apomictic *Paspalum notatum*

**DOI:** 10.1186/s12864-017-3700-z

**Published:** 2017-04-21

**Authors:** Juan Pablo A. Ortiz, Santiago Revale, Lorena A. Siena, Maricel Podio, Luciana Delgado, Juliana Stein, Olivier Leblanc, Silvina C. Pessino

**Affiliations:** 1 Instituto de Investigaciones en Ciencias Agrarias de Rosario (IICAR)-CONICET/Laboratorio de Biología Molecular, Facultad de Ciencias Agrarias, Universidad Nacional de Rosario, Campo Experimental Villarino, Provincia de Santa Fe, Zavalla, S2125ZAA Argentina; 2 Instituto de Agrobiotecnología de Rosario (INDEAR), Ocampo 210 bis, Provincia de Santa Fe, Rosario, 2000 Argentina; 3 UMR 232, Institut de Recherche pour le Développement, Université de Montpellier, Montpellier, 34394 France; 4 Wellcome Trust Centre for Human Genetics, Roosevelt Drive, Oxford, OX3 7BN UK

**Keywords:** Apomixis, Next generation sequencing, Plant reproduction, Sexual reproduction, Transcriptomics

## Abstract

**Background:**

*Paspalum notatum* Flügge is a subtropical grass native to South America, which includes sexual diploid and apomictic polyploid biotypes. In the past decade, a number of apomixis-associated genes were discovered in this species through genetic mapping and differential expression surveys. However, the scarce information on *Paspalum* sequences available in public databanks limited annotations and functional predictions for these candidates.

**Results:**

We used a long-read 454/Roche FLX+ sequencing strategy to produce robust reference transcriptome datasets from florets of sexual and apomictic *Paspalum notatum* genotypes and delivered a list of transcripts showing differential representation in both reproductive types. Raw data originated from floral samples collected from premeiosis to anthesis was assembled in three libraries: *i*) sexual (SEX), *ii*) apomictic (APO) and *iii*) global (SEX + APO). A group of physically-supported *Paspalum* mRNA and EST sequences matched with high level of confidence to both sexual and apomictic libraries. A preliminary trial allowed discovery of the whole set of putative alleles/paralogs corresponding to 23 previously identified apomixis-associated candidate genes. Moreover, a list of 3,732 transcripts and several co-expression and protein –protein interaction networks associated with apomixis were identified.

**Conclusions:**

The use of the 454/Roche FLX+ transcriptome database will allow the detailed characterization of floral alleles/paralogs of apomixis candidate genes identified in prior and future work. Moreover, it was used to reveal additional candidate genes differentially represented in apomictic and sexual flowers. Gene ontology (GO) analyses of this set of transcripts indicated that the main molecular pathways altered in the apomictic genotype correspond to specific biological processes, like biotic and abiotic stress responses, growth, development, cell death and senescence. This data collection will be of interest to the plant reproduction research community and, particularly, to *Paspalum* breeding projects.

**Electronic supplementary material:**

The online version of this article (doi:10.1186/s12864-017-3700-z) contains supplementary material, which is available to authorized users.

## Background

Apomixis (*i.e.* asexual reproduction via seeds) is an intriguing developmental strategy described in more than 400 angiosperm species [1], which results from gaining the ability to bypass the fundamental aspects of sexual reproduction: meiosis and fertilization [2]. Apomixis involves the combination of three fundamental events: lack or failure of meiosis (apomeiosis); fertilization-independent initiation of embryogenesis (parthenogenesis); and formation of functional endosperm either autonomously or after fertilization (pseudogamy) [3]. Apomictic plants are able to produce an offspring genetically identical to the mother. However, some capacity for sexuality is usually maintained; thus, they benefit from using a very sophisticated combination of reproductive strategies, generating diversity and, concurrently, allowing the best fitted individuals to propagate clonally [2, 4, 5].

Apomictic developmental pathways are traditionally classified as either sporophytic or gametophytic [6]. In sporophytic apomicts, a few somatic (2n) cells surrounding the reduced megagametophyte differentiate and form multiple globular-shaped embryos, which develop to maturity by sharing the nutritive endosperm generated from the meiotic legitimate embryo sac. In gametophytic apomicts, unreduced (2n) embryo sacs are formed in the ovule nucellus after a series of mitosis. The formation of these unreduced megagametophytes can follow two mechanistic types, *i. e.* diplospory and apospory, based upon the origin of the precursor cells that ultimately give rise to the mitotically-derived embryo sac: the megaspore mother cell or companion nucellar cells, respectively. Embryo development is fertilization independent, whereas endosperm formation may or may not require fertilization [2, 6].

The ability to produce genetically uniform progeny via seeds is of significant value for its potential in agriculture to fix complex favorable genotypes particularly hybrids expressing heterosis or obtained from wide crosses, to improve breeding programs efficiency in the context of rapidly evolving environmental and social constraints, and promote seed marketing [2, 7, 8]. In fact, the use of apomixis is currently having direct consequences on the breeding of natural apomictic forage grasses of the *Brachiaria* [9–11] and *Paspalum* [12, 13] genera, allowing a significant increase in cattle production in tropical and sub-tropical areas of the Americas. However, major food crops such as maize, rice and wheat are not naturally apomictic and attempts to introduce the trait from wild relatives remain unsuccessful as yet [14, 15]. On the other hand, despite intense research over the past three decades, our current knowledge of the molecular determinants underlying apomictic developments in plants remains largely unknown.

Up to date, two main strategies were applied to the identification of apomixis-relevant genes: one is based on the screening and the analysis of sexual model plants’ mutants, which show components of asexual reproduction; the other is founded on the discovery of candidate genes genetically and/or functionally associated with the trait from natural apomictic species. Both approaches are strongly interrelated and contributed to progress in apomixis research. Genes mimicking elements of apomixis were identified in *Arabidopsis* [16–18] and maize [19, 20]. Moreover, *Arabidopsis* and rice artificial plants producing male and female clonal diploid gametes have already been obtained [21, 22]. On the other hand, several research groups have been focused on the dissection of the genomic region controlling the trait in natural apomictic species [23–38]. In the grasses, the genomic region controlling aposporous apomixis was characterized as a single locus (apomixis controlling locus or ACL) which is often large, complex and recalcitrant to recombination. The deciphering of the genes included within the ACL was attempted by sequencing BAC (bacterial artificial chromosome) clones carrying molecular markers completely linked to aposporous apomixis , such as in *Pennisetum squamulatum* [30], *Cenchrus ciliaris* [39] and *Paspalum simplex* [40, 41], or by performing chromosomal walking from markers cosegregating with the trait, as in *P. notatum* [42]. In all cases, several putative protein-coding regions and a large number of highly repetitive sequences have been detected [30, 39–42]. This approach has recently allowed the identification of a key gene (*BABYBOOM*) related with parthenogenesis in *P. squamulatum* [43] and an apomixis-linked pseudogene (*PsORC3*-like) associated with the control of endosperm formation in *P. simplex* [44]. However, the extent of the non-recombinant ACL and its high gene content complicate the identification of the apomixis trigger/s by positional cloning, turning into one of the major drawbacks for the isolation of apomictic determinant/s by direct genetic approaches. In this scenario, reverse genetic strategies based on the identification of candidates through transcriptomic methods followed by further validation by experimental mapping and functional analyses became central to the apomixis field. Comparative transcriptional surveys were carried out in several grasses, allowing the delivery of an extended list of candidate genes, which could play roles either as prime movers or downstream participants in asexual reproductive development [45–54]. Some of the candidates identified from *Paspalum* are currently under examination in order to investigate their positional and functional linkage with apomixis, *e.g. LORELEI*-like *PNGAP1* [55], *PNSERK* [56] and *PNTGS1*-like [57].

*Paspalum* is one of the largest genera of the *Poacea* family, with nearly 350 representatives [58]. Several of its members form multiploid complexes composed of diploid sexual and polyploid apomictic cytotypes [59]. Over the past five decades, a wealth of information regarding the biology, genetic and reproductive behavior of many *Paspalum* species has been produced. Some of them, like *P. notatum* and *P. simplex*, became valuable models for the study of apomixis, because they concurrently represent systems for mining candidate gene(s) and important forage crops [59]. Natural populations of both species mainly include autoincompatible sexual diploid cytotypes (2n = 2× =20), as well as aposporous apomictic, pseudogamous, self-compatible tetraploid counterparts (2n = 4× = 40) [59]. However, apomictic races hold relatively complex polyploid genomes with 2n = 4× = 40 chromosomes, which are highly heterozygous and of a relatively large size (2C DNA content: 2.2 ± 0.056 pg and 3.0 ± 0.072 pg for *P. notatum* and *P. simplex*, respectively) [60]. The number of characterized sequences deposited in public databases is still limited (127 nucleotide/80 EST (Expressed Sequence Tag)/66 protein sequences for *P. notatum* and 17 nucleotide/7 protein sequences for *P. simplex* at GenBank). Several of them were identified through differential display [48] or cDNA AFLP (Amplified Fragment Length Polymorphism) [53] analyses. Therefore, they are short (most of them 150-400 nt long) and reveal low or no similarity to genes previously characterized in model species. This complicated the annotation of candidates identified in apomixis research projects and the inference of possible functions. Recently, genomic raw data sequences from leaf tissue of *P. simplex* were deposited at NCBI (SRA accession number SRX1149360).

RNA-Seq (RNA sequencing) technologies offer several key advantages over other existing methodologies for characterizing transcriptomes. They are particularly useful for studying non-model organisms, because neither cloning libraries nor any prior knowledge on the species genome are required. Reads derived from RNA-Seq give information about how exons are connected, with longer reads or pair-end short reads revealing connectivity among multiple exons. In addition, RNA-Seq allows characterization of sequence variations in the transcribed regions [61]. Particularly, 454/Roche has become a method of choice for analyzing transcriptomes of non-model organisms, because of its long-read capacity, which makes data more amenable to *de novo* assembly and annotation [62].

The objective of this work was to produce a floral reference transcriptome of apomictic and sexual *P. notatum* plants by using long-read 454/Roche FLX+ next generation sequencing (NGS) technology, which allows sound processing of data with minimal risk of chimeric assembly. The derived transcript database was used to retrieve the full sequences of putative *P. notatum* apomixis candidate genes identified in prior work in this species [48], including all alleles/paralogs expressed in flowers. Moreover, *in silico* comparisons of the apomictic and sexual libraries were used to reveal additional transcripts and several molecular routes that might be involved in asexual development. The availability of this database will considerably broaden our knowledge on the molecular basis of apomixis in this important model genus.

## Results

### RNA sequencing

To characterize the *P. notatum* floral transcriptome, total RNA was extracted from balanced mixes of flowers at different developmental stages: premeiosis, late premeiosis/meiosis, postmeiosis and anthesis. Since this study was aimed at comparing sexual and asexual seed development, flowers were separately collected from two different genotypes with contrasting reproductive modes: C4-4× (2n = 4× = 40; sexual) and Q4117 (2n = 4× = 40; obligate apomict). Two different samples (sexual and apomictic) were created, with all developmental stages evenly represented. After initial quality controls and library preparation, the samples were sequenced using the 454/Roche FLX+ platform (see Materials and Methods). The sexual sample produced 1,367,227 reads of 470.21 ± 175.91 bp average length, which accounted for 642,887,313 total bp (Table 1). The mean GC content was 53.37 ± 9.46%. The apomictic sample produced 1,378,523 reads of 494.88 ± 164.45 bp average length, which accounted for 682,198,061 bp (Table 1). The mean GC content was 52.85 ± 9.13%. Graphic reports on length, GC content and base quality distribution, occurrence of Ns and polyA/T tails, tag sequence checking, sequence duplication, sequence complexity and dinucleotide odds ratios for the sexual and the apomictic samples are provided (see Additional file 1 and Additional file 2).

**Table 1:** Rawdata and *de novo* assemby information for the sexual, apomictic and global libraries

Rawdata	Sexual	Apomictic	Global
Total reads	1.367.227	1.378.523	2.745.750
Total bases	642.887.313	682.198.061	1.325.085.374
Mean sequence length	470,21 ± 175,91	494,88 ± 164,45	482,60 ± 170,70
Minimum length	21	24	21
Maximum length	1.754	1.315	1.754
Mode length	563	536	563
Mean GC content	53,37 ± 9,46	52,85 ± 9,13	53,11 ± 9,30
Minimum GC content	0	0	0
Maximum GC content	96	93	96
Mode GC content	50	51	51
Total contigs	50.503	56.163	79.335
Total bases in contigs	40.566.966	43.448.946	57.550.073
Total isotigs	43.888	47.569	67.617
Total bases in isotigs	51.472.249	56.806.751	86.162.368
Total isogroups	35.430	37.124	48.842
Total isogroups w/ one isotig	30.542	31.137	40.274
Total isotigs w/ one contig	30.455	31.039	40.139

### Transcriptome *de novo* assemblies

**Fig. 1:**
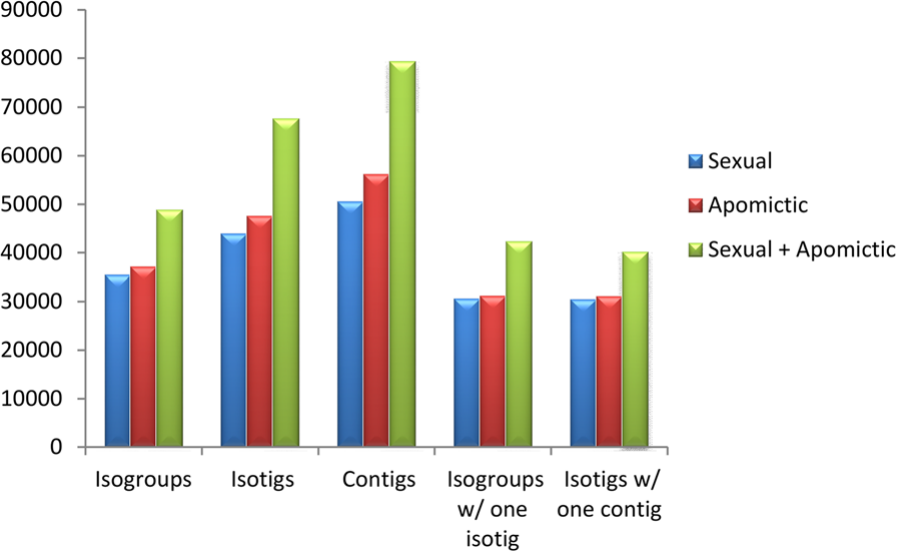
Assemblies derived from apomictic and sexual raw sequence data. The graphic shows the number of isogroups (genes), isotigs (alleles/splice variants), contigs (exons), isogroups with a single isotig (IsoSI) and isogroups with a single contig (IsoSC) obtained after assembly of the sexual, the apomictic and the apomictic + sexual (global) raw sequence data

Approximately 350,000 sequences (26%) corresponding to ribosomal RNAs were detected and consequently filtered from each sample. The average length of the remaining reads (see above) resulted long enough to guarantee a robust assembly (see Methods). The sexual sample (SEX) assembly produced 35,430 isogroups (genes) and 43,888 isotigs (alleles/splice variants) (Table 1, Fig. 1). They derived from a total of 50,503 contigs, with an average size of 803 nt. The number of isogroups with a single isotig was 30,542 and the number of isogroups with more than one isotig resulted 4,888. The number of isotigs with a single contig was 30,455. The apomictic sample (APO) assembly produced 37,124 isogroups and 47,569 isotigs. A total of 56,163 contigs with an average size of 773 nt were detected. The number of isogroups with a single isotig was 31,137 and the number of isogroups with more than one isotig resulted 5,987. The number of isotigs with a single contig was 31,039. Next, a global assembly was built after bulking the reads from both the sexual and the apomictic samples, and then following the same methodology as described above. In this case, 48,842 isogroups and 67,617 isotigs were assembled. A total of 79,335 contigs with an average size of 725 nt were detected. The number of isogroups with a single isotig was 40,274. The number of isogroups with more than one isotig was 8,568. The number of isotigs with a single contig was 40,139 (Table 1, Fig. 1).

### Validation of the isotigs assembly

In order to validate the assembly of the isotigs obtained in both genotypes, we carried out a comparison using 24 mRNA and 80 EST sequences of *P. notatum*, previously identified and deposited at NCBI (July 2016). These sequences were used as queries in BLAST (Basic Local Alignment Search Tool) searches against the isotigs generated from the SEX and APO assemblies. All 24 mRNA sequences (average length of 839.04 ± 509.65 bp) identified hits in both assemblies with E-value (expect value) < 0.00005 (see Additional file 3). On average, 2.6 and 3.25 mRNA sequences matched with the same isotig in the sexual and the apomictic databases, respectively, indicating that several of the previously identified sequences represent alleles or paralogs of individual genes. The percentage of identity (%ID) of the alignments in the sexual and apomictic libraries ranged from 91.459 – 100% (average 98.442% ± 2.011) and 94.408 – 100% (average 98.602% ± 1.438), respectively. The length of the alignments in the sexual and apomictic databases varied from 281 to 1220 bp (average 693.395 ± 331.599 bp) and from 296 to 1245 bp (average 698.667 ± 324.769 bp), respectively. The score values in the sexual and apomictic databases varied from 370 to 2245 (average 1230 ± 607.839) and from 453 to 2274 (average 1240.041 ± 588.458), respectively. Besides, the analysis of ESTs sequences (average length 215.53 bp) showed that 81.250% and 83.750% of them matched with E-value < 0.00005 in the sexual and apomictic databases, respectively (see Additional file 3). The average %ID value and the average alignment length in the sexual and apomictic libraries were 96,900 (±3.070)/96.689% (±3.331) and 125.747 bp (±85.487)/132.225 bp (±86.190), respectively. Moreover, the mean query coverage fraction for the sexual and apomictic databases was 0.6439 (±0.3457) and 0.7816 (±0.2466), respectively.

**Fig. 2:**
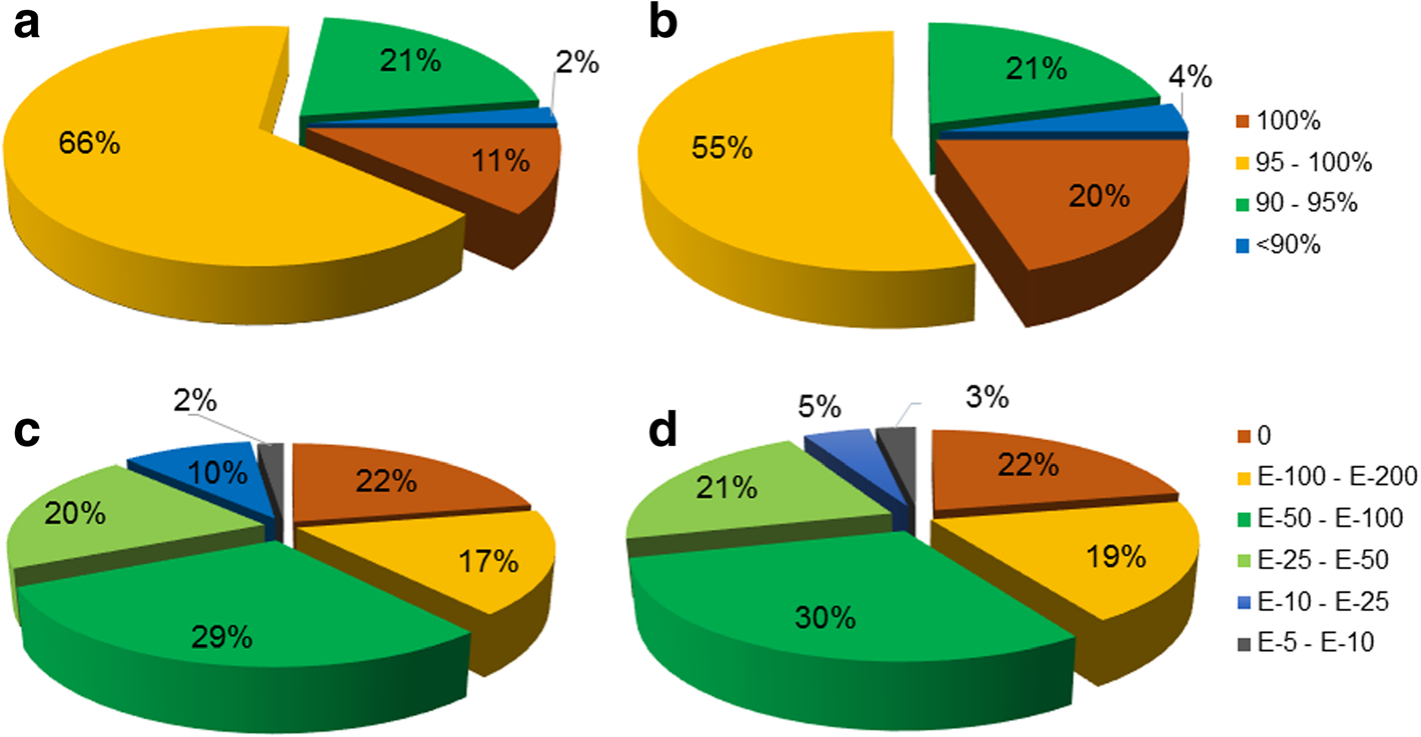
Validation of 454 libraries using BLAST search *P. notatum* mRNA (*n =* 80) and EST (n24) sequences deposited at NCBI were used as BLASTN queries against the 43,888 and 47,569 assembled isotigs from sexual (**a**, **c**) and apomictic (**b**, **d**) 454/Roche transcriptome libraries. **a**-**b**: Distribution of similarities values for the BLAST hits. **c**-**d**: E-value distribution of BLAST hits at a threshold E-value ≤ 10e^-5^

In order to better characterize our BLAST results, we calculated the distribution of similarities and E-values obtained with all queries (mRNA + ESTs). The sexual library analysis showed that 87% of the sequences had %ID greater than 95%, and only 2% revealed values lower than 90% (Fig. 2a and b). Likewise, similarity values in the apomictic database showed that 75% of the sequences had %ID values higher than 95% and only 4% had less than 90% (Fig. 2a and b). The analyses of distribution of the E-values in the sexual library showed that 88% of the sequences aligned with E-values lower than e^-50^, and only 2% with values between e^-5^ and e^-10^. Similar values were obtained in the apomictic database (92% of the sequences aligned with E-values lower than e^-50^ and 3% showed values between e^-5^ and e^-10^) (Fig. 2c and d). These results indicated that previously identified *P. notatum* sequences expressed in reproductive tissues are well represented in both SEX and APO assemblies, therefore making these database reliable tools for the identification and the characterization of novel genes.

### Characterization of the floral transcriptome landscape of *Paspalum notatum*

**Fig. 3:**
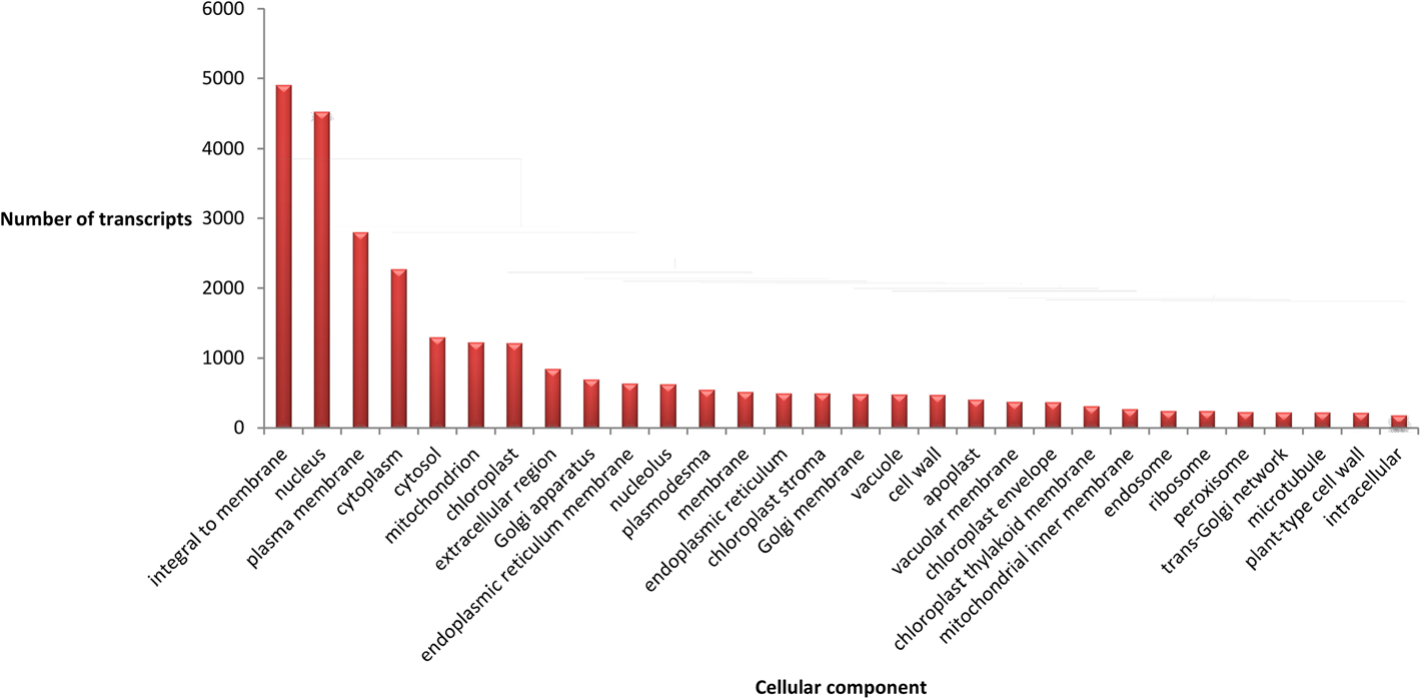
Cell components represented in the *P. notatum* floral transcriptome global assembly. The number of transcript units for the 30 most represented ontology terms is displayed

**Fig. 4:**
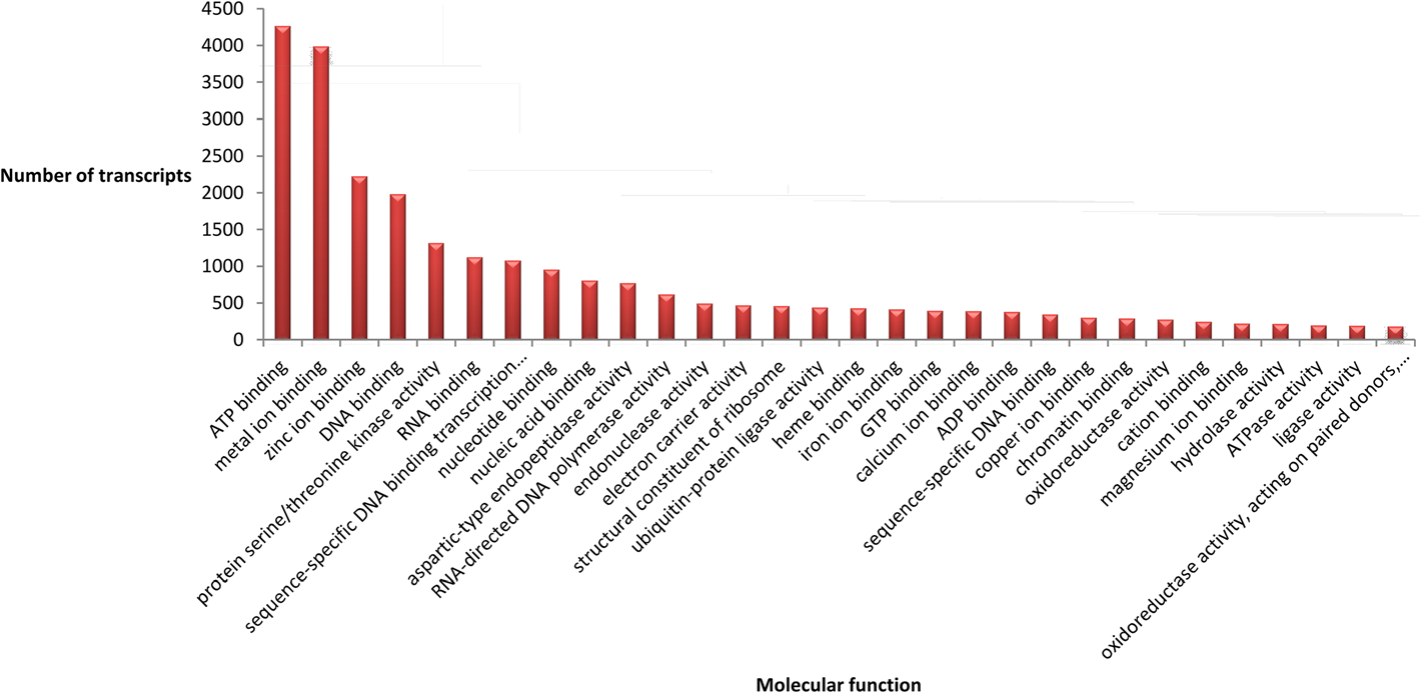
Molecular functions represented in the *P. notatum* floral transcriptome global assembly. The number of transcript units corresponding to the 30 most represented ontology terms is displayed

**Fig. 5:**
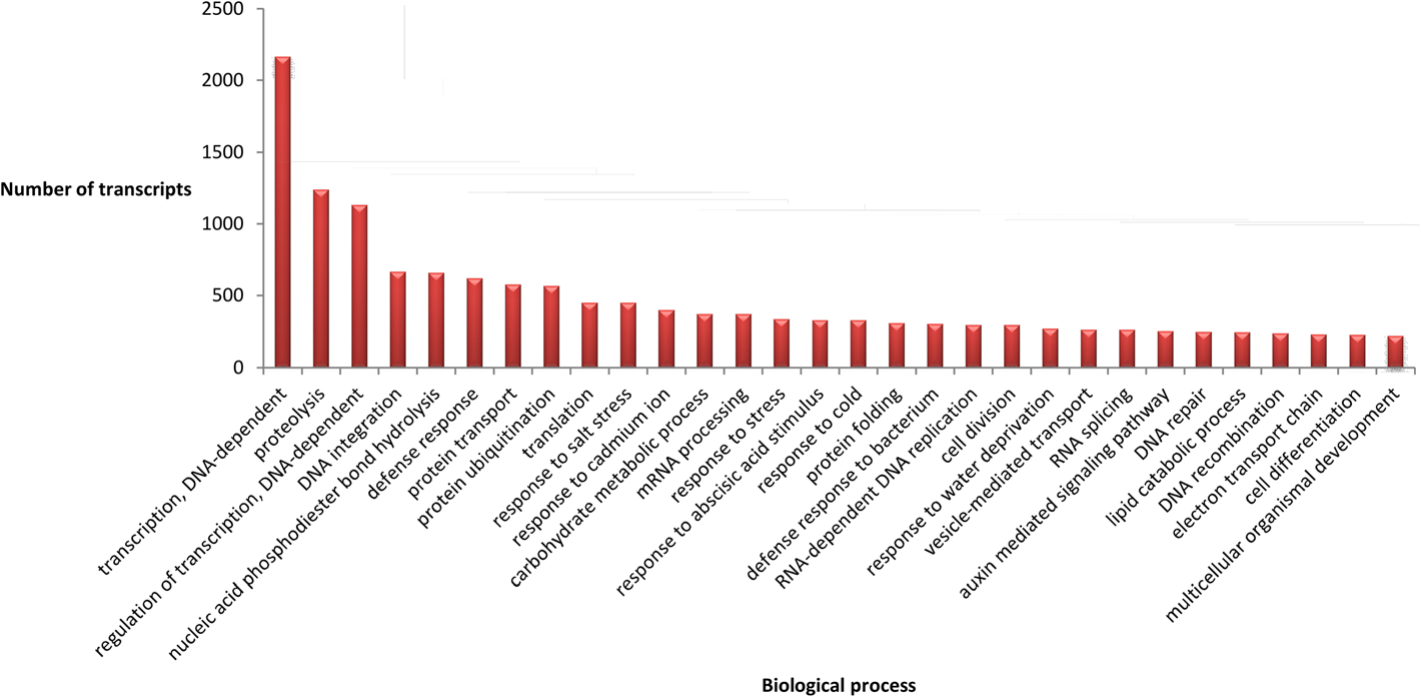
Biological processes represented in the *P. notatum* floral transcriptome floral assembly. The number of transcript units corresponding to the 30 most represented ontology terms is displayed

A list of the annotated isotigs produced from the global assembly is shown in Additional file 4. Our analyses revealed that in *P. notatum* flowers the products of protein-coding transcripts are distributed in at least 772 different cellular locations, performing 2,102 molecular functions and participating in 3,375 biological processes. These numbers correspond to the different reproductive developmental stages (from premeiosis to anthesis) of both sexual and apomictic reproductive modes found in the species. The 30 most represented cellular components, molecular functions and biological processes are shown in Fig. 3, 4 and 5, respectively. These major classes accounted for more than three quarters of all the cellular component units (27,685 out of 36,483), half of all the molecular component units (25,406 out of 44,699) and almost one-third of the biological processes units (14,273 out of 46,996). Future research and /or breeding programs focused in specific gene classes could benefit from the use of this analysis, since it could provide a glimpse of the number of genes that can be investigated in a target category by using these libraries (~330 genes related to cold response, a relevant set to breeding programs; ~220 genes related to cell differentiation, a gene group of interest to apomixis research).

### Full-sequence recovery of *P. notatum* apomixis candidate genes

In order to retrieve the full cDNA sequences of apomixis-associated candidate genes reported in prior analyses, we decided to focus on a set of gene fragments identified by Laspina et al. (2008) [48] using differential display (DD). This procedure allows the isolation of sequence tags of 100-400 bp, mainly at the transcript 3’ ends. Therefore, DD isolated fragments frequently corresponded to short, non-conserved 3’ UTR (untranslated region), rendering the identification of homologs in the databases difficult. Here we used 65 of these sequence fragments as queries to retrieve the full cDNAs from the *P. notatum* global library. Twenty-four candidates matched apo and/or sex isotigs (N5, N7, N12, N15, N17, N18, N20, N26, N43, N46, N51, N54, N56, N58, N60, N69, N95, N98, N99, N108, N114, N115, N116, N119) (see Additional file 5). The full cDNA sequences of all detectable alleles corresponding to these genes were recovered. The number of alleles/splice variants (isotigs) per gene (isogroup) varied from 1 to 8 (average 1.960 ± 1.822) and the length of the transcripts varied from 471 to 8,696 (average 2,924.42 ± 2,198.18). Six (6) candidates (25% of the tagged genes) identified as “unknown” (n) in the previous analysis (N7, N17, N58, N99, N116, N119) [48] could be annotated. They corresponded to genes associated with a trafficking protein, a thaumatin-like protein, a copia protein, a nuclear pre-mRNA domain-containing protein 1B, a carotenoid cleavage dioxygenase and an unknown function protein. Besides, 2 candidates (N60, N108) changed to a different annotation due to a more accurate similarity search (a Leishmanolysin-like peptidase and FAR1-RELATED protein, respectively).

### Transcript representation comparison between apomictic and sexual libraries

**Fig. 6:**
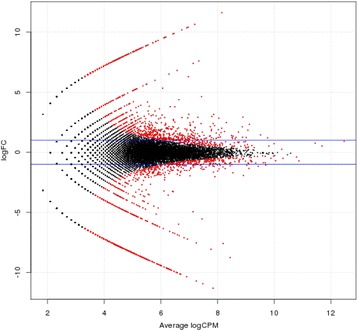
Gene expression pairwise comparison between sexual and apomictic *Paspalum notatum* samples. For each isogroup, the log fold change (logFC) was plotted against the log of counts per million mapped reads (logCPM). Black dots: non-significant differential expression. Red dots: significant differential expression (*p* value < 0.01). Positive logFC: upregulation in apomictic samples. Negative logFC: upregulation in sexual samples

The number of raw reads mapping onto the global assembly was computed for both the apomictic (APO) and the sexual (SEX) samples. Pairwise comparisons allowed the identification of a number of potentially differentially expressed genes (Fig 6). A subset of 3,732 isotigs showing differential expression patterns at raw p-value (probability value) < 0.01 and logFC (logarithm transformed fold change) > 3 was selected as candidates putatively associated with the reproductive mode (see Additional file 6). Out of these, 2,066 were overexpressed in the apomictic plant, while 1,666 were upregulated in the sexual plant. These results suggest that numerous genes poorly expressed or silent during sexual reproduction are transcriptionally activated in ovules of apomictic plants. The first 1,784 transcripts display adjusted p-values (FDR or false discovery rate) < 0.01 (Additional file 6).

### Identification of molecular routes potentially altered during apomixis

**Fig. 7:**
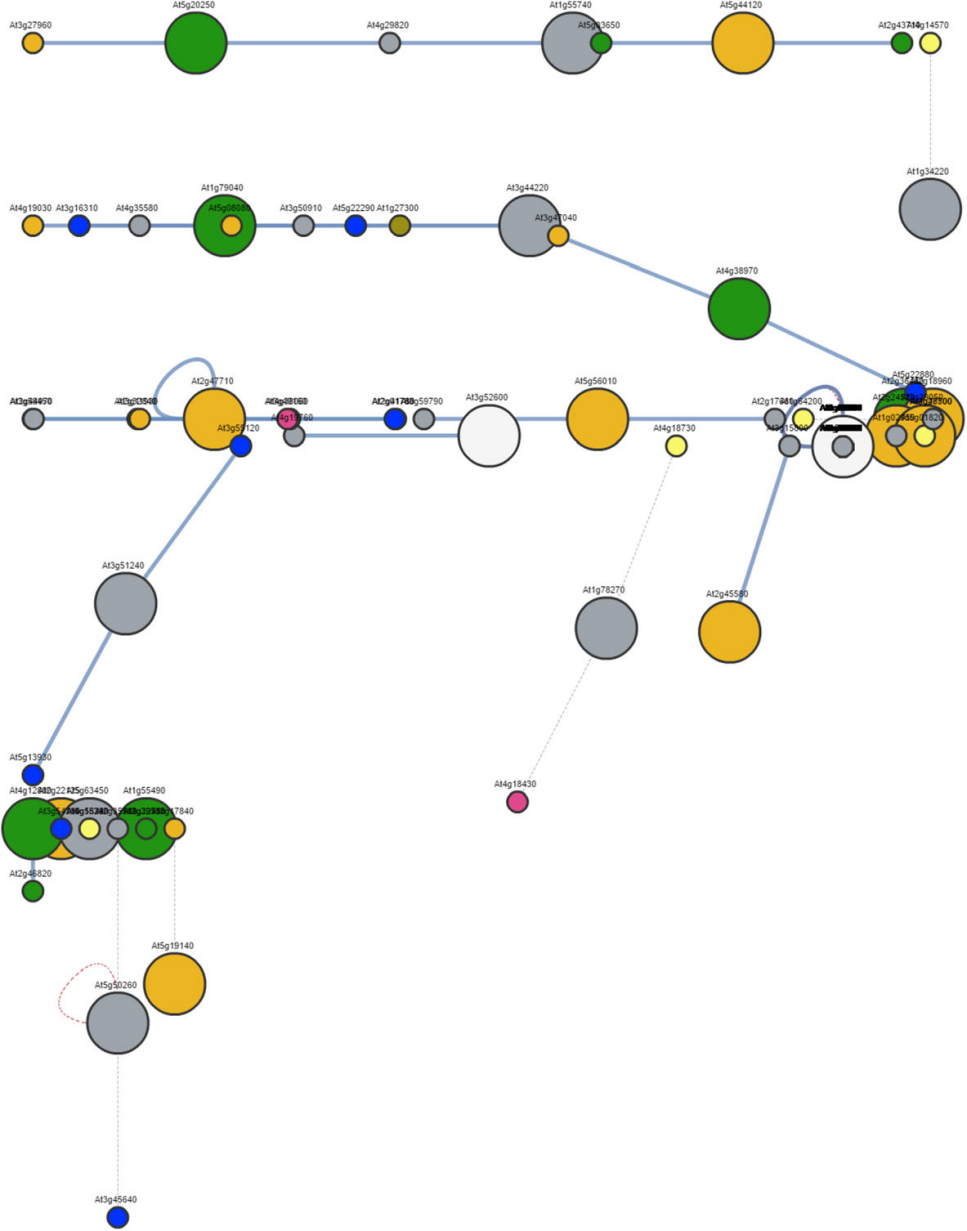
Graphical representation of candidate protein-protein interaction networks involved in apomictic reproduction. Networks were generated with the BAR *Arabidopsis* interaction viewer (Geisler-Lee et al. 2007), by loading the putative Arabidopsis orthologs of the 200 top ranked candidates differentially expressed between the sexual and apomictic 454 libraries. Nodes represent proteins with the following localizations: green: chloroplast; pink: cytoplasm; yellow: vacuole; orange: plasma membrane; blue: nucleus; grey: unknown. Edges indicate the interactions. Light blue bold lines mark the experimentally determined interactions

In order to identify putative molecular routes associated with the apomictic development in *P. notatum*, we hypothesized that the significantly differentially expressed candidates described above will show a trend to group into common plant molecular expression networks. The putative *Arabidopsis* orthologs corresponding to the differentially expressed candidates were retrieved from TAIR (see Additional file 7). Next, we used PLANEX to detect molecular networks putatively altered during apomixis using the 200 top ranked differential candidates (those with the lowest false discovery rate values). PLANEX clusters containing the highest numbers of modulated genes were: 213, 56 and 51 (with 12, 7 and 5 modulated genes, respectively); 62, 176 and 354 (with 4 potentially modulated genes each); and 75, 78, 79, 83, 167, 247, 308 and 310 (with 3 potentially modulated genes each). These groups were mainly related with the following ontology terms: photosynthesis, ion transport, ribonucleotide metabolic processes, protein complex biogenesis and assembly, monosaccharide catabolism, translation, gene expression, small molecule catabolic processes, proteolysis, protein transport, targeting, localization and folding, DNA replication, cell wall modification, aminoacid metabolism and regulation of RAS activity, among others. Moreover, processing of these same 200 top ranked candidates with the BAR *Arabidopsis* interaction viewer (http://bar.utoronto.ca) [63] allowed the identification of 5 protein-protein interaction clusters with at least one member altered during apomictic development (Fig. 7). These apomixis-related protein interaction networks contained a total of 59 *Arabidopsis* genes (see Additional file 8) that are associated with the following biological processes: biotic and abiotic stress (clusters 1, 2, 3, 4), cell cycle control (clusters 1, 4), development (clusters 1, 2, 3, 4), cell death and senescence (clusters 2, 3, 5), growth (clusters 2, 3, 4), and post embryonic lethality (cluster 5). The identification of such networks is essential for future detailed characterization of the molecular routes involved in apomixis.

## Discussion

In the last few years, dozens of gene fragments associated with apomixis were identified in *Paspalum* spp. through mapping analysis [26, 27, 29, 42, 64, 65], BAC sequencing [40, 41], differential display [45, 48] or cDNA AFLP [53]. However, these approaches were compromised either by the structural organization complexity of the apomixis locus or by the relatively short size of candidate fragments, making difficult the recovery of reliable sequence information for further annotation and functional characterization. Therefore, the main goal of this study was to generate a global, annotated reference database for the reproductive transcriptome of *Paspalum* and, to achieve this, the use of a long-read RNAseq technique allowing the establishment of robust assemblies was mandatory.

Here, apomictic and the sexual 454/Roche libraries were compared to discover genes involved in the switch between both reproductive modes. A total of 35,430/ 37,124 and 48,842 different genes (isogroups) were identified in the sexual, the apomictic and the global assembly, respectively. The highest number of genes (isogroups) detected in the global assembly is probably reflecting both a deeper coverage of the transcriptome (by random sequencing of a biological duplicate) and the presence of genes that are specifically expressed in either of the samples, which were originated from plants with different genotypes and reproductive modes. Besides, the number of alleles/splice variants (isotigs) detected in the sexual genotype C4–4× (43,888) was lower than that observed in the apomictic one Q4117 (47,569). This result is in agreement with the plants biological origin: C4–4× is a dihaploid generated by colchicine duplication of a natural diploid [66]; by contrast, Q4117 is a natural highly heterozygous tetraploid genotype. Therefore, a lower genetic variation could be anticipated in C4-4× with respect to Q4117. Validation of our assemblies by comparison with physically supported sequences indicated that both databases extensively cover the *P. notatum* floral transcriptome. Moreover, alignment scores suggested their high potential for full length transcript identification. The database may allow the recovery of allele/splice variants corresponding to dozens of apomixis and ploidy response candidate genes that had been identified and verified in prior works through PCR (polymerase chain reaction)-based approaches [45, 48, 53, 67]. It can also be used for the generation of EST-SSR (Expressed Sequence Tag-Simple Sequence Repeat) markers covering the *Paspalum* spp. genome for mapping experiments and breeding.

We conducted GO analysis in the whole reference assembly in order to reveal the location and nature of the biological processes operating in florets. The apomictic and sexual reads were mapped on this general annotated assembly as a common reference. The GO annotation and subsequent general analysis of major classes provided a general view of gene activity in reproductive organs. Moreover, its use might greatly simplify the comparison of the molecular routes involved in species displaying different apomixis mechanisms. According to our results, in *P. notatum* flowers, the products of protein-coding transcripts are distributed in at least 772 different cellular locations, performing 2,102 molecular functions and participating in 3,375 biological processes. Note that these numbers correspond to different reproductive developmental stages, and also include the two possible reproductive modes via seeds that alternatively occur (apomixis and sexuality). Moreover, the biological samples used consisted of whole flowers (spikelets), which comprise the raquis, glumes, lemma, palea, ovary and anthers. Therefore, the whole set of transcripts characterized here derives from a variety of cell types including somatic cells and male and female reproductive cells from premeiosis to anthesis. Accordingly, the database will be very useful to identify any transcript expressed in *Paspalum* flowers at detectable levels. However, the spatial and temporal specificity of expression will need to be assessed by using additional experiments based on *in situ* hybridization, specific promoter-directed markers expression and/or tissue- or cell-type q-PCR.

One of the major weaknesses of molecular reproductive research in *Paspalum* was the need to carry out laborious RACE (Random Amplification of cDNA Ends) experiments in order to isolate the full sequences of candidates genes expressed in flowers. RACE amplification experiments were in fact conducted successfully for several candidates [55–57], but the recovery of full genic sequences turned out very difficult and time consuming, especially for complex or long transcripts. Moreover, the characterization of all allelic/splice variants expressed in flowers was virtually impracticable. Here, through the use of next generation sequencing, we successfully recovered the full cDNA sequences of 24 differential display (DD) fragments, including several detectable alleles/splice variants, therefore validating the value of our database for the detailed characterization of specific gene family members. From the 65 DD sequence segments used as queries, only 24 matched the apo and sexual 454 isotigs. Lack of detection of the remaining sequences could be explained from the emergence of false positives in DD analysis and/or poor 454 sequencing coverage. However, 20 of the sequence segments used as queries showed no BLAST hits in the sequence databases of plant species and several of them were amplified from internal parts of the target transcripts (displaying two random decamers located at the edges). Therefore, they might correspond to different parts of the same transcript, leading to an overestimation of the rate of sequences undetected in the 454/Roche database.

The public availability of a global database of transcripts expressed during reproductive development will also be of invaluable benefit for harnessing important target traits in *Paspalum* breeding research. The use of apomixis is currently having a direct impact on the breeding of natural *Paspalum* species [59]. Among other species of the genus, *P. notatum* and *P. dilatatum* are the most widely cultivated forage grasses. The specific objectives of the breeding are directed to the enhancement of cold tolerance and cool-season growth, seed yield, grazing/biotic stress resistance and nutritive value [59]. Advanced breeding programs were conducted under two different approaches: 1) germplasm collection, evaluation, selection, multiplication of the best ecotypes, and release of elite genotypes as new apomictic cultivars; and 2) hybridization using sexual mother plants and apomictic male progenitors, followed by the selection of superior full apomictic progeny hybrids, which breed true due to its clonal reproductive mode. The availability of the sequence database reported here would make possible the characterization of numerous genes responsible for important metabolic/biological pathways and their transfer to different genetic backgrounds by traditional breeding or genetic engineering. Moreover, we recently established a biolistic transformation platform for tetraploid *P. notatum* in our laboratory [68], a tool that will certainly benefit from the sequence data we generated here to engineer the expression of genes related to reproduction and seed yield. Further characterization of leaf and root transcriptomes would be also desirable in order to provide additional useful information.

While the apomictic, sexual and global 454 reference libraries are useful to rescue the full sequences of a considerable number of candidate genes, its use as a tool to reveal differential expression is more limited, because contrasting representation can be masked by heterochronic expression in sexual and apomictic samples and/or differential expression being restricted to a very particular developmental stage and or individual cells. In order to achieve accurate assessment of differential expression, deeper coverage approaches should be used, i.e. Illumina sequencing. However, the construction of a reference transcriptome is a pre-requisite to Illumina (short-read) sequencing, in order to reach a sound assembly in these complex non-model polyploidy systems. Therefore, although the estimation of differential expression is considered preliminary and needs further validation, it revealed a number candidate genes and cluster networks that are potentially altered during apomictic development. Many of the top ranked genes in the differential expression list are included in protein-protein interaction clusters related to abiotic and biotic stress response, growth, development, cell death and senescence. Particularly, the detection of numerous candidates related with the first category supports previous hypothesis pointing to the participation of stress response pathways on meiosis initiation [69] and the early preparatory events ahead of apomeiotic transition [70], as well as the influence of environmental factors and polyploidization genomic shocks on the expressivity of facultative apomixis [71]. Once particular pathways associated with apomixis are identified, a scrutiny of the correlation associations within these networks and the physical location of particular candidates within the ACL have the potential to reveal the nature of the genes controlling both apomeiotic transition and parthenogenesis. Therefore, the systematic use of the information provided in this report will contribute to accelerate the discovery of the triggers of apomixis and to the future harnessing of the trait.

## Conclusions

The controlled use of apomixis in plant breeding programs requires a detailed characterization of its molecular basis. The present study identified the floral transcriptome components for sexual and aposporous *P. notatum* genotypes along all reproductive stages from premeiosis to anthesis, providing full sequences for numerous reproductive candidate genes. Moreover, it detected expression differences between the apomictic and the sexual biotypes. While this evidence provides hints on the molecular pathways involved in apomixis development, further research concerning genomic and functional characterization is needed for revealing the nature of its genetic determinants.

## Methods

### Plant material

The *P. notatum* genotypes characterized in this work were: 1) a natural apomictic genotype, Q4117 (2n = 4× = 40), originated from Southern Brazil [72]; and 2) an artificial double diploid sexual genotype, C4–4× (2n = 4× = 40), experimentally obtained from chromosome duplication of a sexual diploid plant by colchicine treatment [66]. Vegetative replicates of these plants are being maintained in experimental plots at IBONE, CONICET-UNNE (Instituto de Botánica del Nordeste, Corrientes, Argentina) and IICAR, CONICET-UNR (Instituto de Investigaciones en Ciencias Agrarias de Rosario, Rosario, Argentina).

### RNA-seq library construction and 454/Roche FLX+ sequencing

Inflorescences from both apomictic (Q4117) and sexual (C4–4×) genotypes were collected at four developmental stages following a procedure and the reproductive calendar reported by Laspina et al. (2008) [48]: early premeiosis (0), late premeiosis/meiosis (I/II), postmeiosis (III/IV/V/VI) and anthesis (VI). Spikelets at each stage of development were separated from the rachis, bulked in equal parts (depending on developmental stage they represented) and frozen in liquid nitrogen. Total RNA was extracted with SV RNA Total Isolation Kit (Promega). Samples were quantified with the Quant-iT RiboGreen RNA Reagent and Kit (Invitrogen). Messenger RNAs were purified and quantified with Dynabeads (Invitrogen) and Quant-iT RiboGreen RNA (Invitrogen), respectively. The RNA quality was evaluated with RNA 6000 Pico chip (Agilent Bioanalyzer 2100). Two-hundreds (200) ng of purified mRNA was subjected to chemical fragmentation, double-strand cDNA synthesis, end repair and phosphorylation, adapter ligation and purification with AMPureXP (Beckman Coulter), according to the recommended by the RL FLX+ (Roche) cDNA Rapid Library Preparation Method Manual (May 2011). The fragmentation quality was controlled in a RNA 6000 Pico Chip (Agilent Bioanalyzer 2100). The libraries were quantified by qPCR using a Kapa Library Quantification Kit 454 Roche Lib-L, as indicated by the manufacturers. Different library dilutions were analyzed in triplicate to allow inclusion in the standard curve. A library titration was carried out by doing an emulsion PCR in small-scale (SV-emPCR), using the GS Titanium SV-emPCR Kit Lib-L v2 (Roche) and following the protocol detailed in emPCR Amplification Method Manual – Lib L SV (May 2011). The emulsion PCR was carry out at large scale (LV-emPCR), expecting an enrichment percentage of 5-20%, using the GS Titanium LV-emPCR Kit Lib-L v2 (Roche) and following the protocol detailed in emPCR Amplification Method Manual – Lib L LV (May 2011). Then, the samples were sequenced using a GS FLX+ Roche sequencer. A whole titanium plate was used for each library, according to the protocol described in Sequencing Method Manual FLX+ Roche (June 2013).

### Sequence data analysis and assembly

Low quality reads, adapters and primers were eliminated by using PRINSEQ [73]. Sequences corresponding to rRNAs were identified and removed using two comparison methods: BLASTn [74] and HMMer [75] against custom rRNA databases and HMM models, respectively. *De novo* assemblies were carried out on data produced from the sexual sample (SEX: sex assemblies), from the apomictic sample (APO: apo assemblies) and from both samples together (GLOBAL: global assemblies), with Newbler v2.8 for cDNA with urt option.

### Validation of the assembly by BLAST against *P. notatum* sequences

Twenty-four (24) mRNA and 80 EST nucleotide sequences of *P. notatum* deposited at the NCBI GenBank (July 2016) were downloaded and used as queries for nucleotide BLAST search against the 43,888 and 47,569 isotigs of the sexual and apomictic library, respectively. An E-value = 10^-5^ was used as cut off threshold for matching sequences. Identity percentages, score values, alignments length, query coverage (align length/query length), and E-value distributions were used as parameters to validate the sequences represented in both libraries.

### Gene expression comparison between the apomictic and sexual libraries

Pairwise comparisons of gene expression between sexual and apomictic *P. notatum* floral genes were carried out using the EdgeR package [76]. The log fold change (logFC) and the log of counts per million mapped reads (logCPM) were calculated for comparing expression levels.

### Sequence annotation and classification

Functional annotation and analysis of the global (apo + sex) *de novo* transcriptome was conducted by using Trinotate (http://trinotate.github.io/) [77]. Based on the GO annotation, the global (apo + sex) transcripts were grouped into classes included within the following categories: molecular function, cellular component and biological pathway. In order to classify the transcripts, individual units of transcript-ontology classes were determined. Three ontology categories were used: 1) cellular component; 2) molecular function; and 3) biological process. Then, all units sharing the same ontology category were grouped and all isotigs sharing the same class within the same category were counted. Thus, a single transcript can be alternatively associated with none, one or several classes within each category, according to the automated classification made by Trinotate.

### Identification of sequences potentially associated with apomixis

A subset of sequences differentially represented in the sexual and the apomictic samples was selected using a *p*-value ≤ 0.01 and logFCs ≥ 3. A BLASTx analysis against the *Arabidopsis* Araport11 peptide database (https://www.arabidopsis.org) allowed the identification of putative Arabidopsis orthologs corresponding to the differentially expressed *P. notatum* candidates. Then, these sequences were used to interrogate the co-expression database PLANEX (http://planex.plantbioinformatics.org/co-expression-search) in order to reveal molecular networks, which alteration could be involved in apomictic reproduction. Discovery of common ontology terms to the genes included in each particular cluster was done with GOTermfinder [78]. Protein-protein interaction networks were investigated by using the *Arabidopsis* Interaction Viewer at the BAR (The Bioanalytic Resource for Plant Biology) webpage (http://bar.utoronto.ca/).

## Additional files

Additional file 1: Graphic report on length, GC content and base quality distribution, occurrence of Ns and polyA/T tails, tag sequence checking, sequence duplication, sequence complexity and dinucleotide odds ratios for the sexual sample. (PDF 300 kb)

Additional file 2: Graphic reports on length, GC content and base quality distribution, occurrence of Ns and polyA/T tails, tag sequence checking, sequence duplication, sequence complexity and dinucleotide odds ratios for the apomictic sample. (PDF 301 kb)

Additional file 3: Comparison of mRNA sequences of *P. notatum* deposited at Genbank (July 2016) with the assembled isotigs of the 454/Roche sexual and apomictic libraries. (XLSX 36 kb)

Additional file 4: Annotation of isotigs originated from the global assembly. (XLSX 7203 kb)

Additional file 5: BLAST analysis of cDNA sequences associated to apomixis identified by Laspina et al. (2008) against the 454/Roche global library. (DOC 37 kb)

Additional file 6: Transcripts differentially represented at the apomictic and sexual libraries at p-value ≤ 0.01 and logFC ≥ 3. (XLSX 888 kb)

Additional file 7: Arabidopsis orthologs corresponding to all differentially expressed candidates. (XLSX 834 kb)

Additional file 8: Protein-protein interaction clusters affected during the transition from sexuality to apomixis. (DOC 37 kb)

## Abbreviations

ACL: Apomixis controlling locus
AFLP: Amplified fragment length polymorphism
APO: Apomictic
BAC: Bacterial artificial chromosome
BLAST: Basic local alignment search tool
DD: Differential display
EST: Expressed sequence tag
EST-SSR: Expressed sequence tag-simple sequence repeat
E-value: Expect value
FDR: False discovery rate
GO: Gene ontology
ID: Identity
logCPM: Logarithm transformed counts per million mapped reads
logFC: Logarithm transformed fold change
NGS: Next generation sequencing
PCR: Polymerase chain reaction
*p*-value: Probability value
RACE: (Random amplification of cDNA ends)
RNA-Seq: RNA-sequencing
SEX: Sexual
UTR: Untranslated region
