# Supplementary material for: A reference floral transcriptome of sexual and apomictic Paspalum notatum
Source: BMC Genomics. 2017 Apr 21;18:318. doi: 10.1186/s12864-017-3700-z (PMC5399859; doi:10.1186/s12864-017-3700-z)
Supplement: Supplementary file 1 — Graphic report on length, GC content and base quality distribution, occurrence of Ns and polyA/T tails, tag sequence checking, sequence duplication, sequence complexity and dinucleotide odds ratios for the sexual sample. (PDF 300 kb) [file 12864_2017_3700_MOESM1_ESM.pdf]

PRINSEQ-graphs v0.6 HTML Report

[Generated: 02/05/2014 10:54:20]

Input Information

Input file(s):	**pnsex.reads.fna and pnsex.reads.qvl**
Input format(s):	**FASTA and QUAL**
# Sequences:	**1,367,227**
Total bases:	**642,887,313**

Length Distribution

Mean sequence length:	**470.21 ± 175.91 bp**
Minimum length:	**21 bp**
Maximum length:	**1,754 bp**
Length range:	**1,734 bp**
Mode length:	**563 bp with 4,938 sequences**

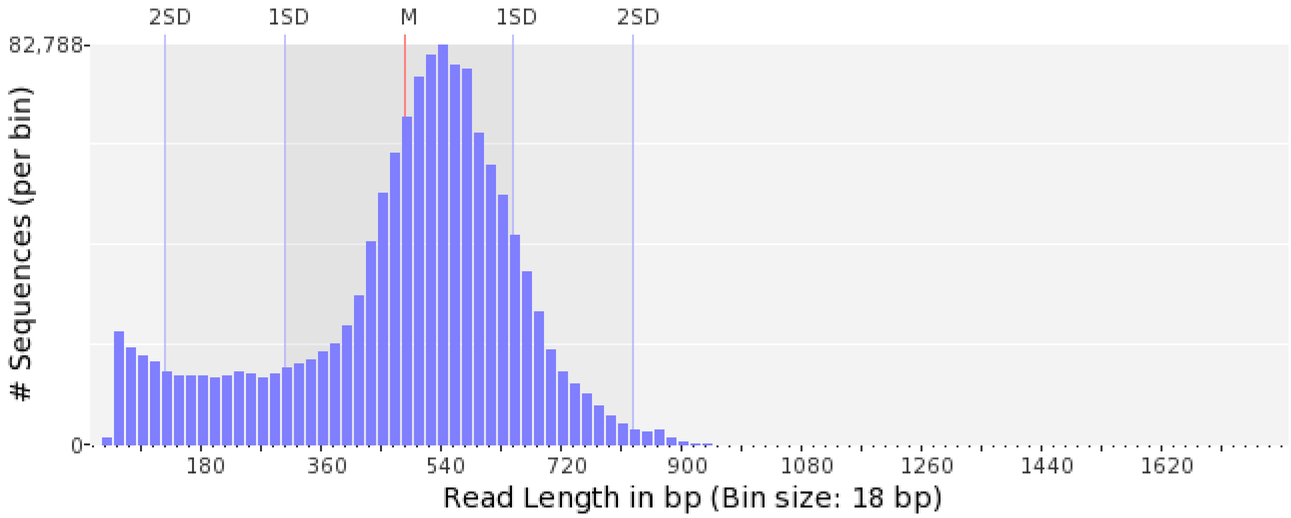

GC Content Distribution

Mean GC content:	**53.37 ± 9.46 %**
Minimum GC content:	**0 %**
Maximum GC content:	**96 %**
GC content range:	**97 %**
Mode GC content:	**50 % with 74,892 sequences**

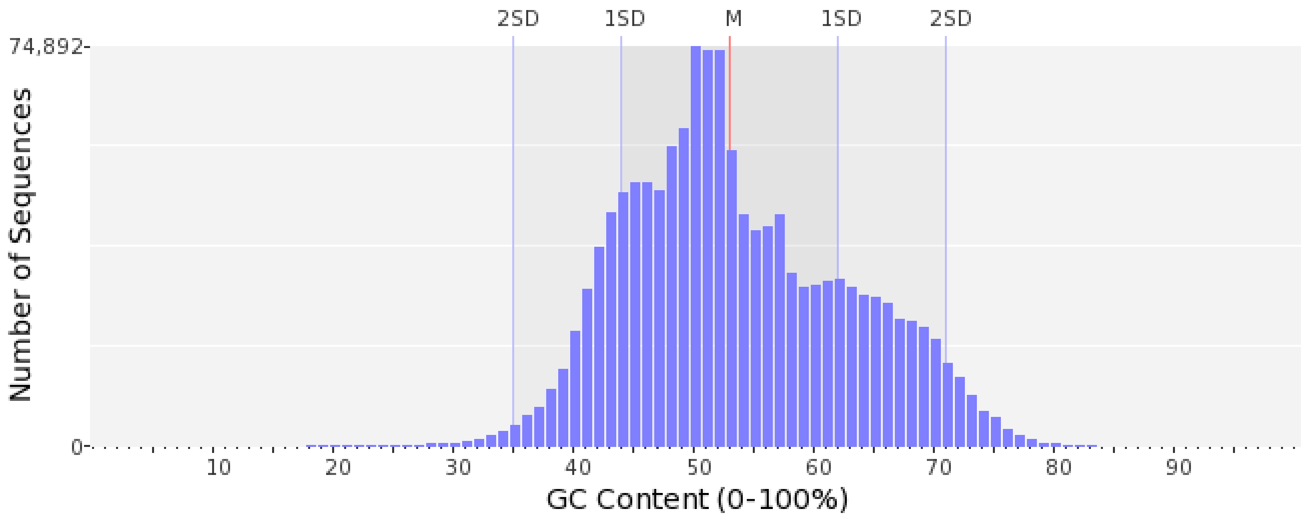

Base Quality Distribution

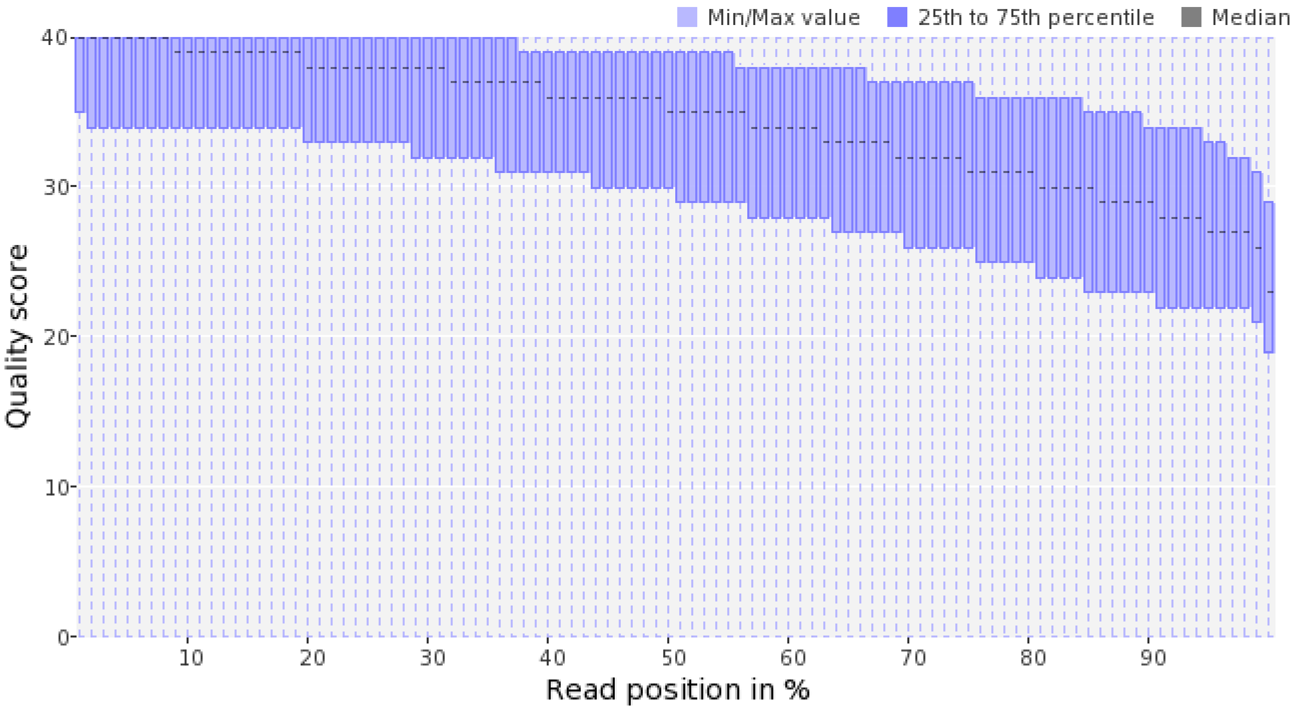

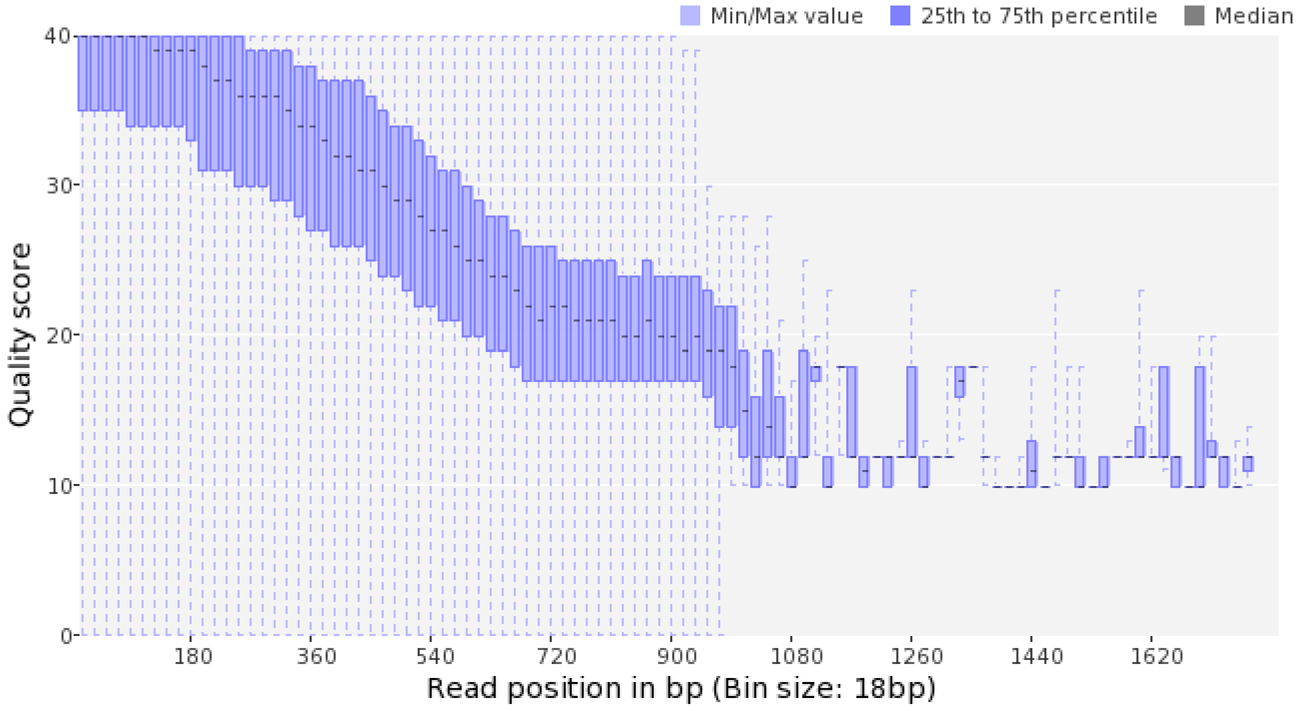

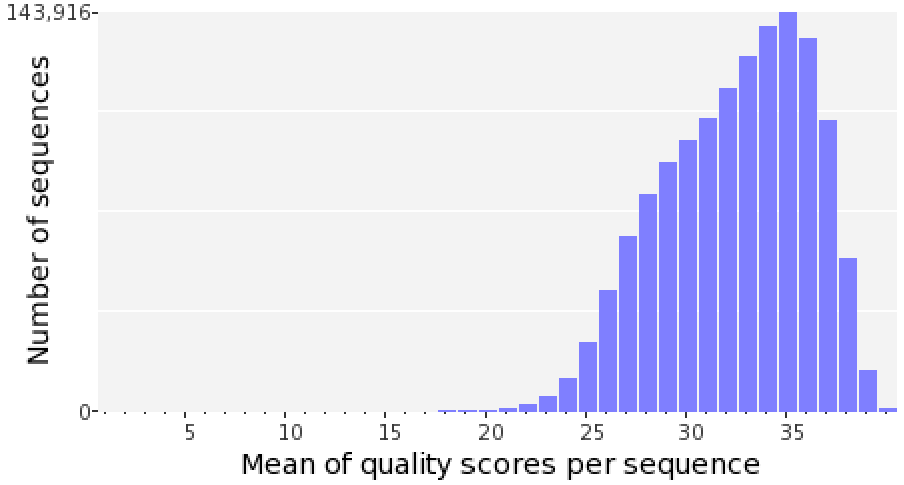

Occurence of N

Sequences with N:	**64,804 (4.74 %)**
Max percentage of Ns per sequence:	**39 %**

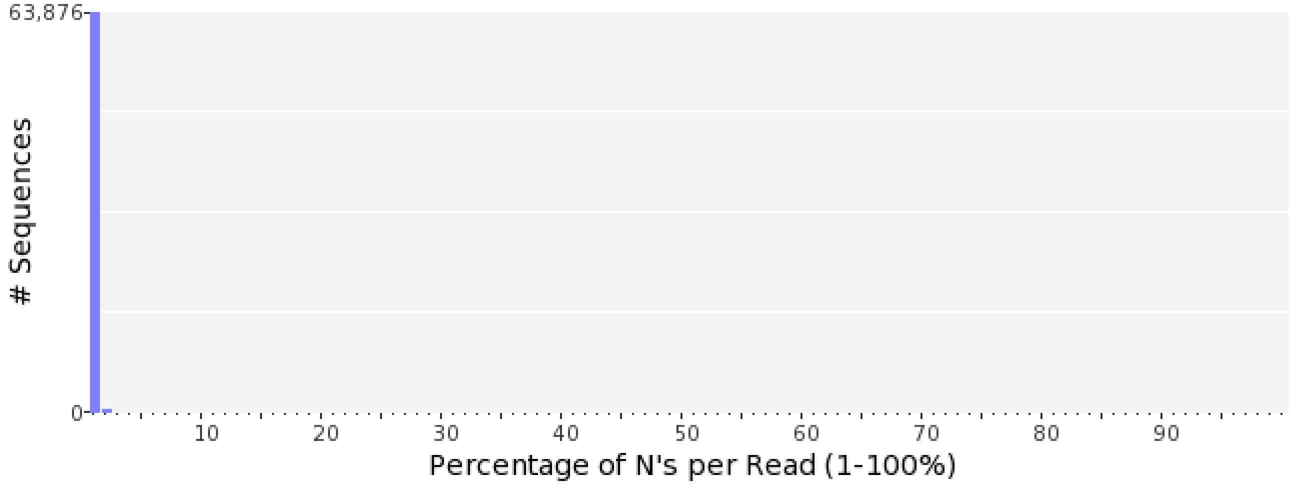

Poly-A/T Tails

	5'-end	3'-end
Sequences with tail:	**4,282 (0.31 %)**	**11,422 (0.84 %)**
Maximum tail length:	**133**	**408**

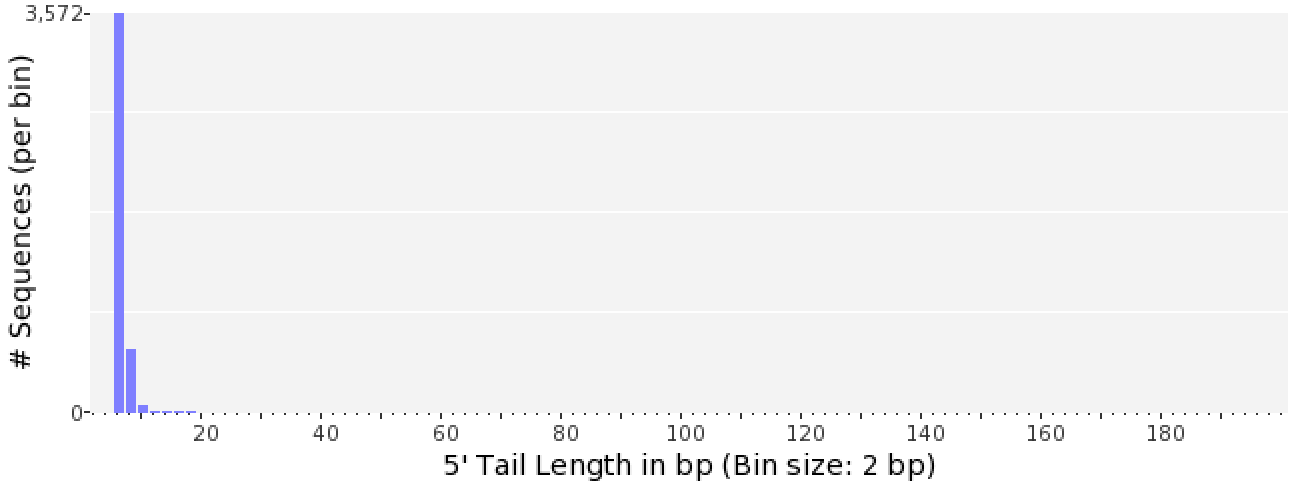

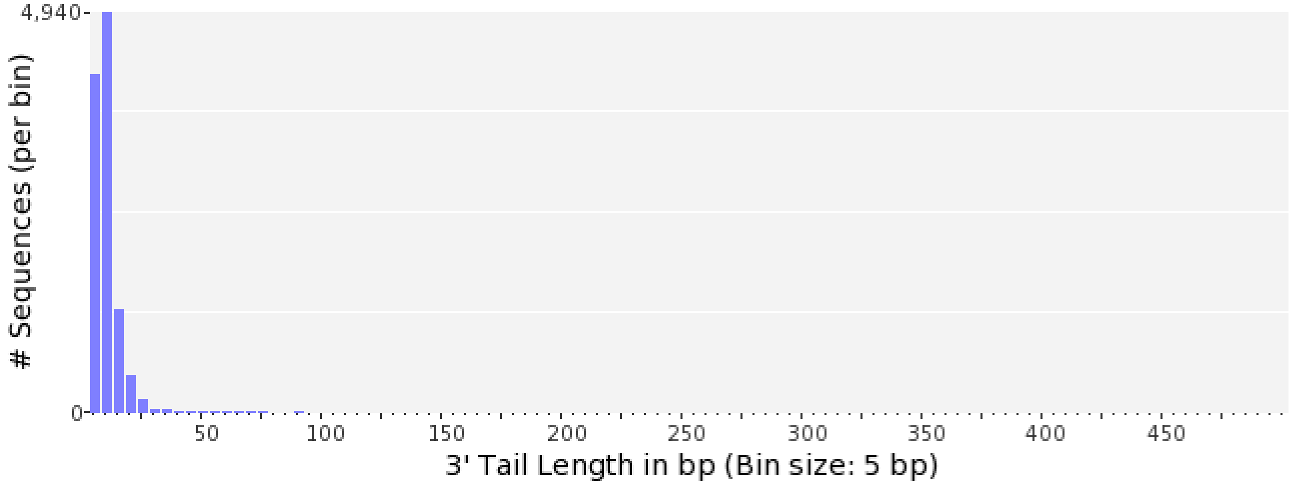

Tag Sequence Check

	5'-end	3'-end
Probability of tag sequence:	**0 %**	**9 %**
GSMIDs or RLMIDs:	**none**	

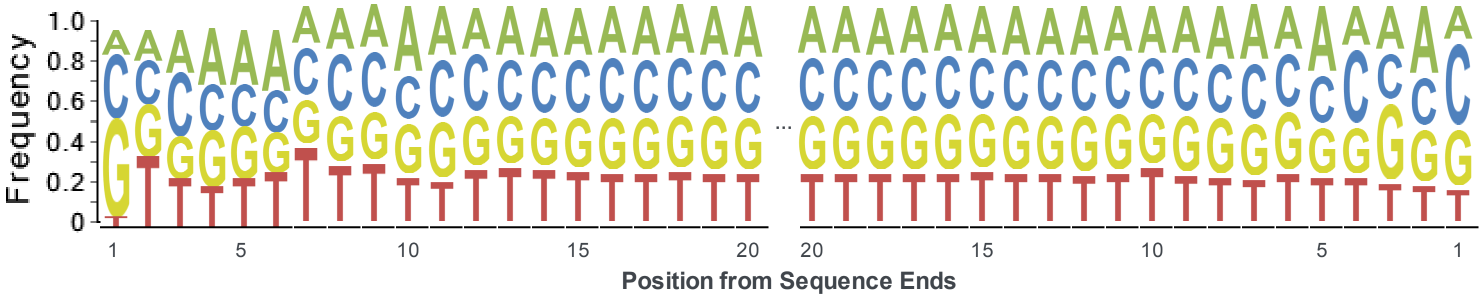

Sequence Duplication

	# Sequences	Max duplicates
Exact duplicates:	**48,319 (3.53 %)**	**172**
Exact duplicates with reverse complements:	**147 (0.01 %)**	**1**
5' duplicates	**125,348 (9.17 %)**	**14**
3' duplicates	**26,108 (1.91 %)**	**45**
5'/3' duplicates with reverse complements	**14,605 (1.07 %)**	**3**
Total:	**214,527 (15.69 %)**	**-**

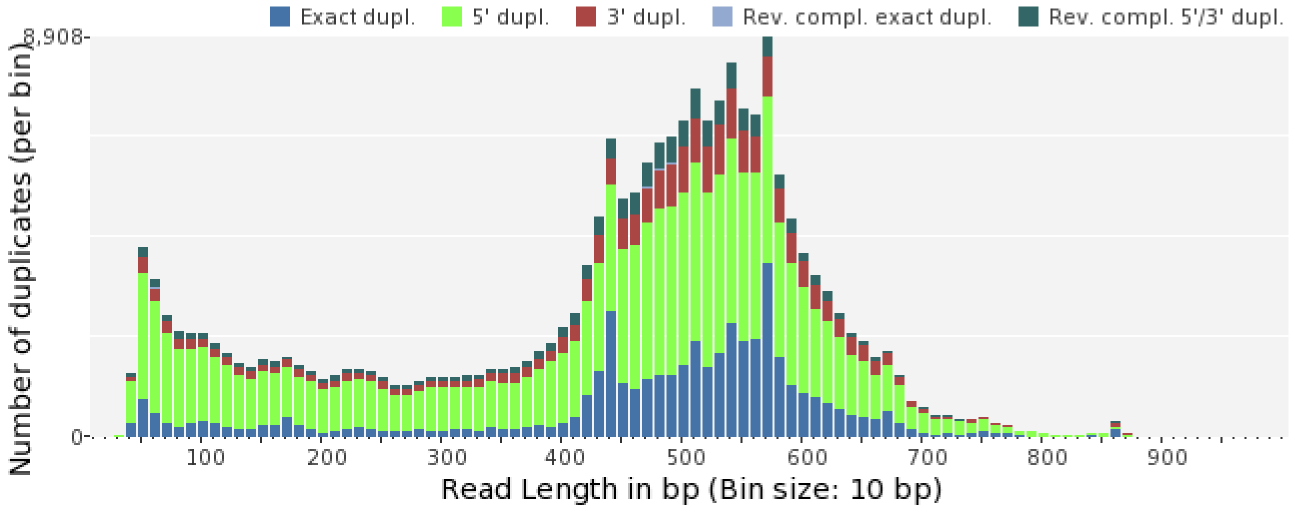

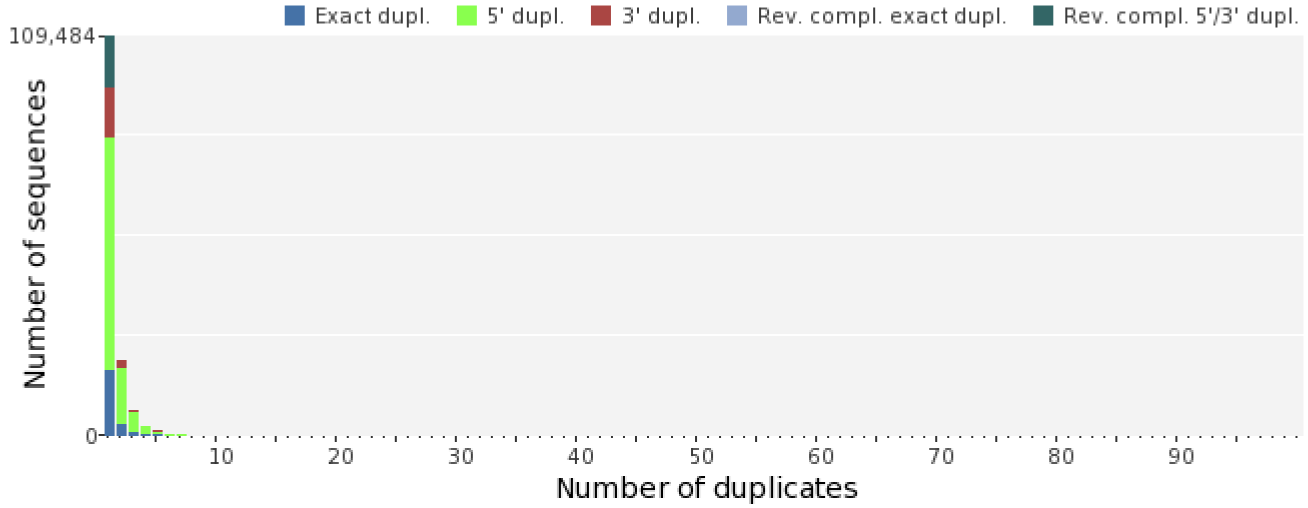

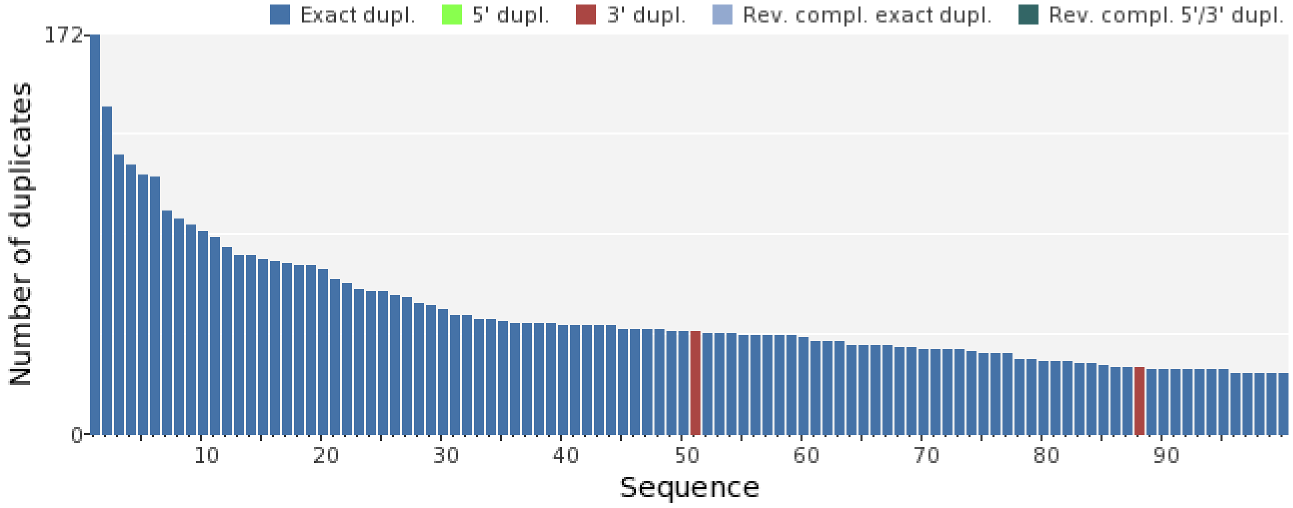

Sequence Complexity

	Value	Sequence
Minimum DUST score:	**0**	ACGCCCTCGACCTATTCTCAACTTAATAGGTA

Maximum DUST score:	**87**	TGTTTTTTTTTTTTTTTTTTTTTTTTTTTTTNTTTTTTTTTTTTTTTTTTTTTTTTTTTT<br>TTTTTTTTTTTTTTTTTTTTTTTTTTTTTTTTTTTTTTTTTTTTTTTTTTTTTTTTTTTT<br>TTTTTTTTTTTTTTTTTTTTTTTTNTTTTTTTTTTTTTTTTTTTTTTTNTTTTTTTTTTT<br>TTTTTTTTTTTTTTTTTTTTTTTTTTTTTTTNTTTTTTTTTTTTTT
Minimum Entropy value:	**6**	TGTTTTTTTTTTTTTTTTTTTTTTTTTTTTTNTTTTTTTTTTTTTTTTTTTTTTTTTTTT<br>TTTTTTTTTTTTTTTTTTTTTTTTTTTTTTTTTTTTTTTTTTTTTTTTTTTTTTTTTTTT<br>TTTTTTTTTTTTTTTTTTTTTTTTNTTTTTTTTTTTTTTTTTTTTTTTNTTTTTTTTTTT<br>TTTTTTTTTTTTTTTTTTTTTTTTTTTTTTTNTTTTTTTTTTTTTT
Maximum Entropy value:	**100**	CTCACTTCCCGCTGCGGGAAGTGAG

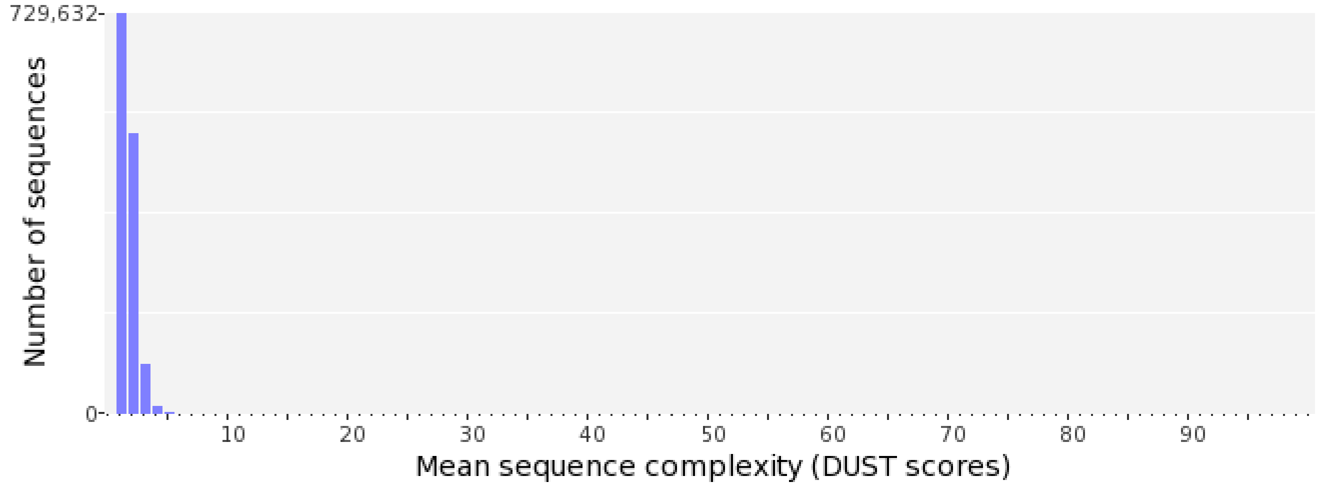

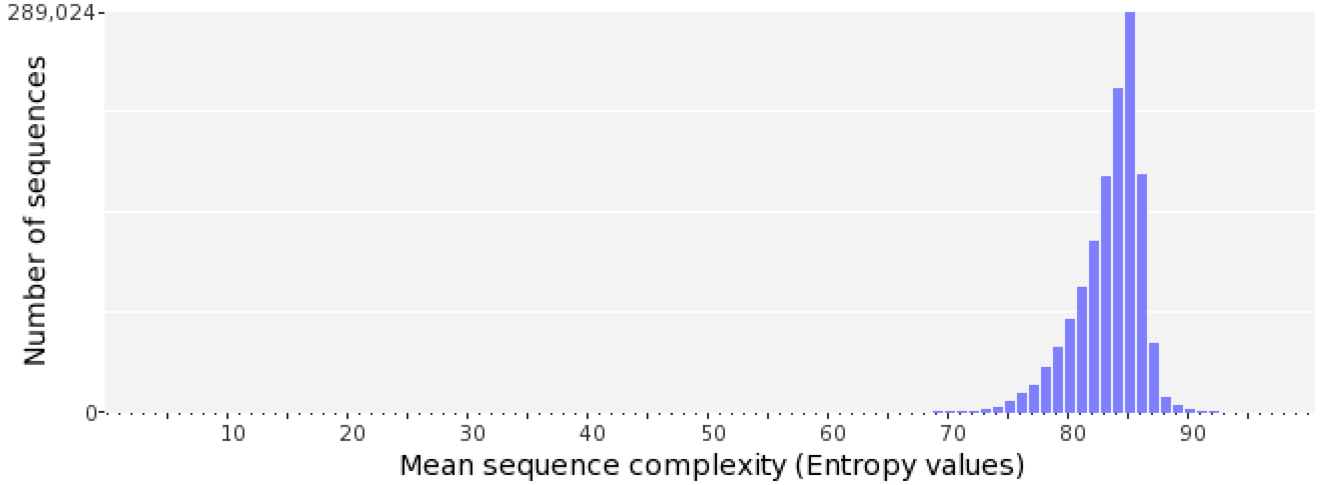

Dinucleotide Odds Ratios

	AA/TT	AC/GT	AG/CT	AT	CA/TG	CC/GG	CG	GA/TC	GC	TA
Odds ratio	**1.0582**	**0.8883**	**1.0717**	**0.9810**	**1.1347**	**0.9699**	**0.8031**	**1.1030**	**1.0606**	**0.6634**

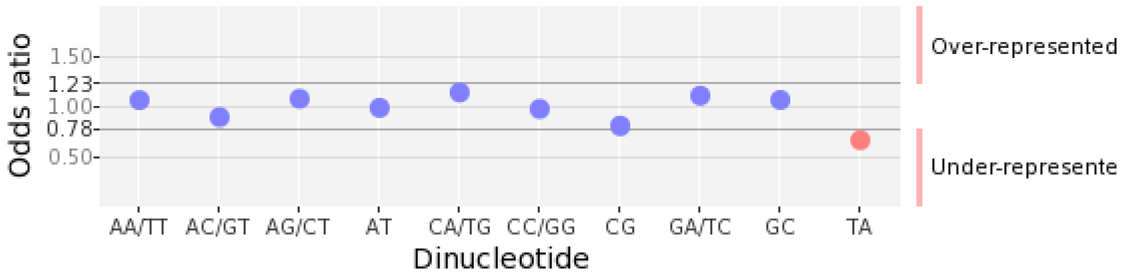

M

0 - User input 1 - Human (fecal) 2 - Mouse (fecal) 3 - Marine (coastal)
4 - Marine (open ocean) 5 - Marine (estuary) 6 - Fish (gut) 7 - Fish (slime)

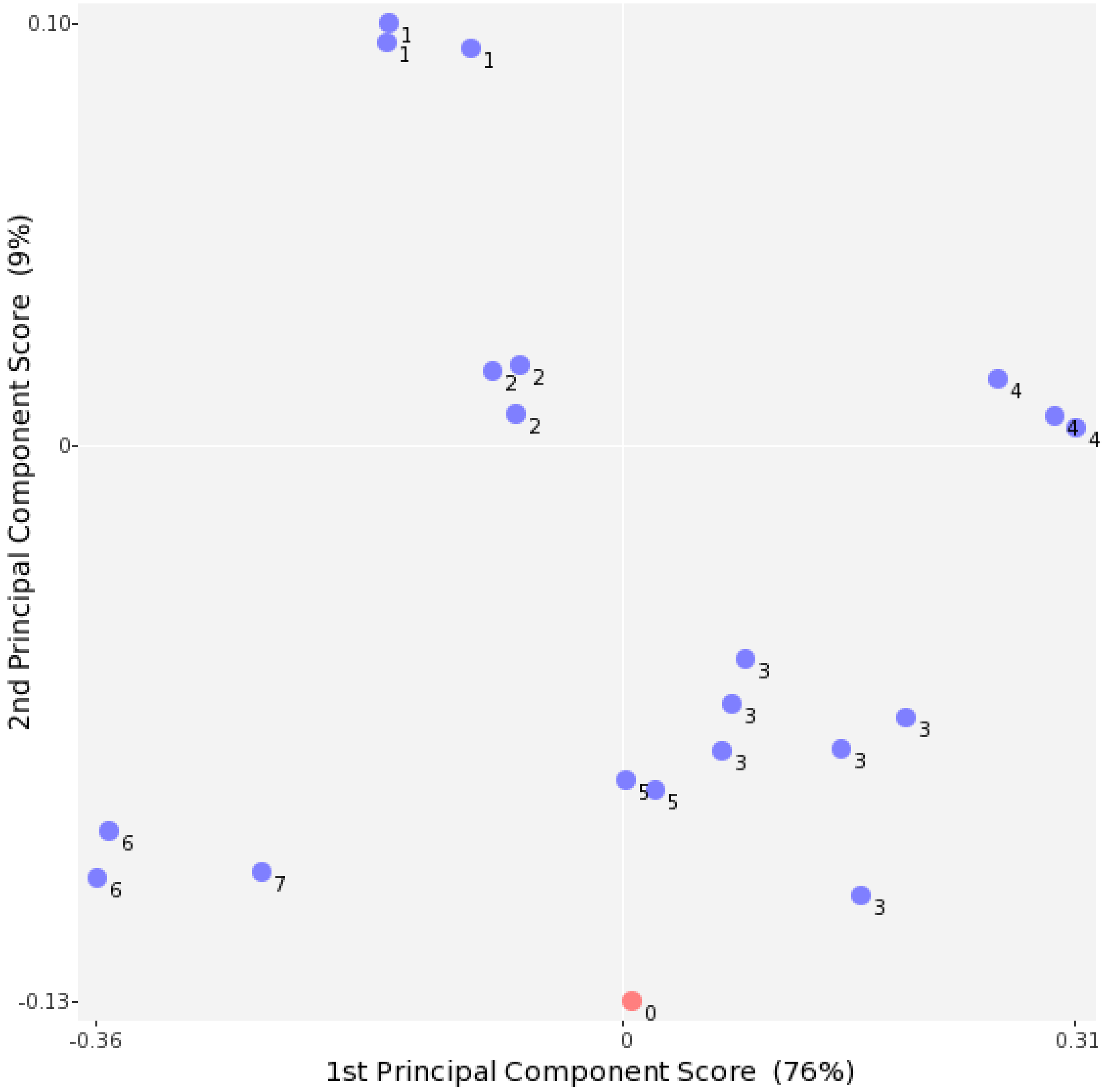

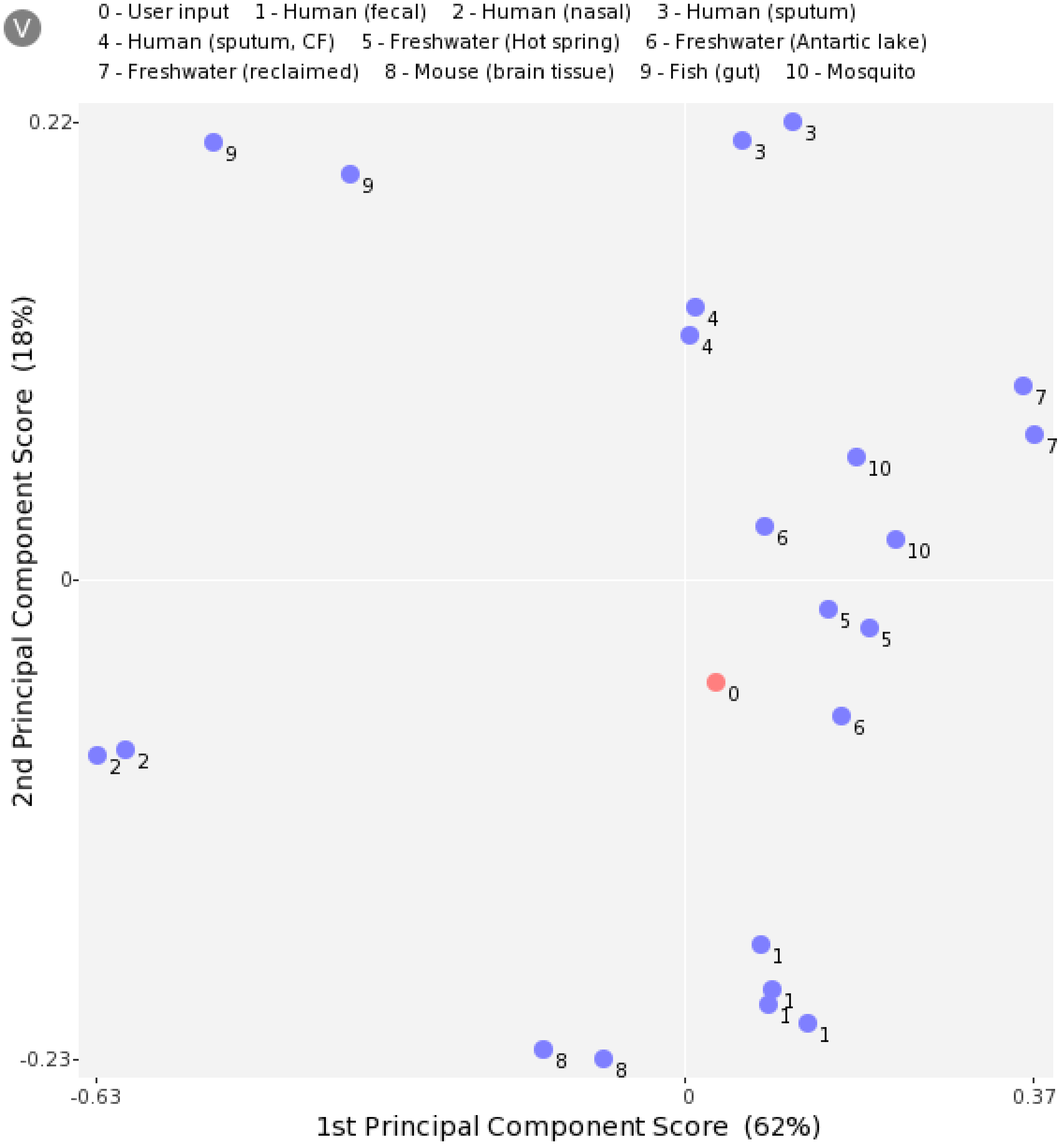
V
0 - User input
1 - Human (fecal)
2 - Human (nasal)
3 - Human (sputum)
4 - Human (sputum, CF)
5 - Freshwater (Hot spring)
6 - Freshwater (Antartic lake)
7 - Freshwater (reclaimed)
8 - Mouse (brain tissue)
9 - Fish (gut)
10 - Mosquito
2nd Principal Component Score (18%)
1st Principal Component Score (62%)
0.22
0
-0.23
-0.63
0
0.37
