# Supplementary material for: A reference floral transcriptome of sexual and apomictic Paspalum notatum
Source: BMC Genomics. 2017 Apr 21;18:318. doi: 10.1186/s12864-017-3700-z (PMC5399859; doi:10.1186/s12864-017-3700-z)
Supplement: Supplementary file 2 — Graphic reports on length, GC content and base quality distribution, occurrence of Ns and polyA/T tails, tag sequence checking, sequence duplication, sequence complexity and dinucleotide odds ratios for the apomictic sample. (PDF 301 kb) [file 12864_2017_3700_MOESM2_ESM.pdf]

PRINSEQ-graphs v0.6 HTML Report

[Generated: 02/05/2014 11:49:57]

Input Information

Input file(s):	**pnapo.reads.fna and pnapo.reads.qvl**
Input format(s):	**FASTA and QUAL**
# Sequences:	**1,378,523**
Total bases:	**682,198,061**

Length Distribution

Mean sequence length:	**494.88 ± 164.45 bp**
Minimum length:	**24 bp**
Maximum length:	**1,315 bp**
Length range:	**1,292 bp**
Mode length:	**536 bp with 5,081 sequences**

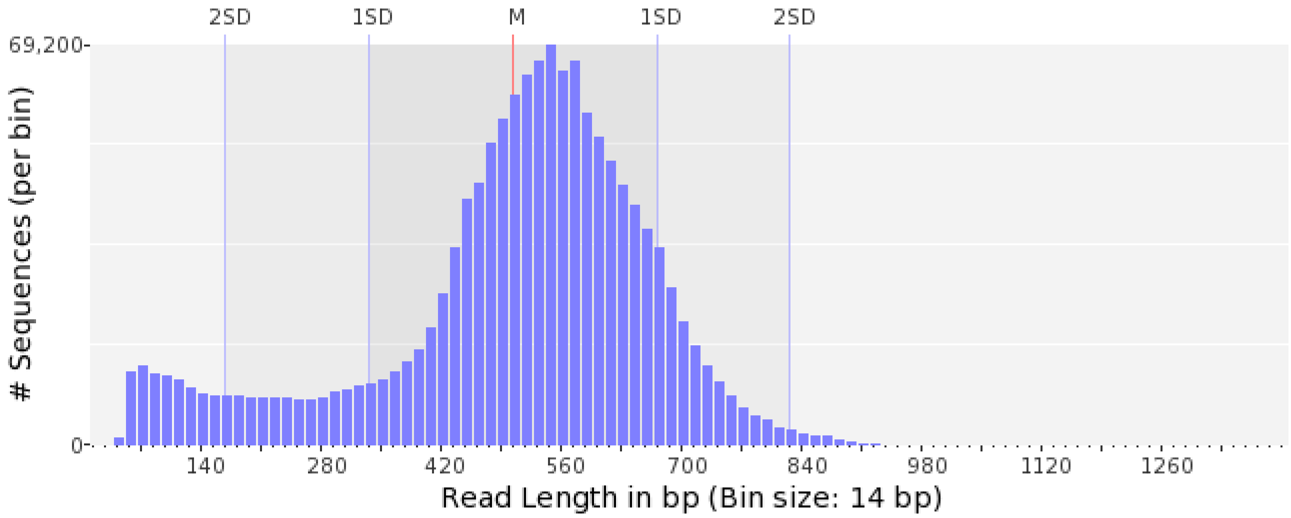

GC Content Distribution

Mean GC content:	**52.85 ± 9.13 %**
Minimum GC content:	**0 %**
Maximum GC content:	**93 %**
GC content range:	**94 %**
Mode GC content:	**51 % with 83,094 sequences**

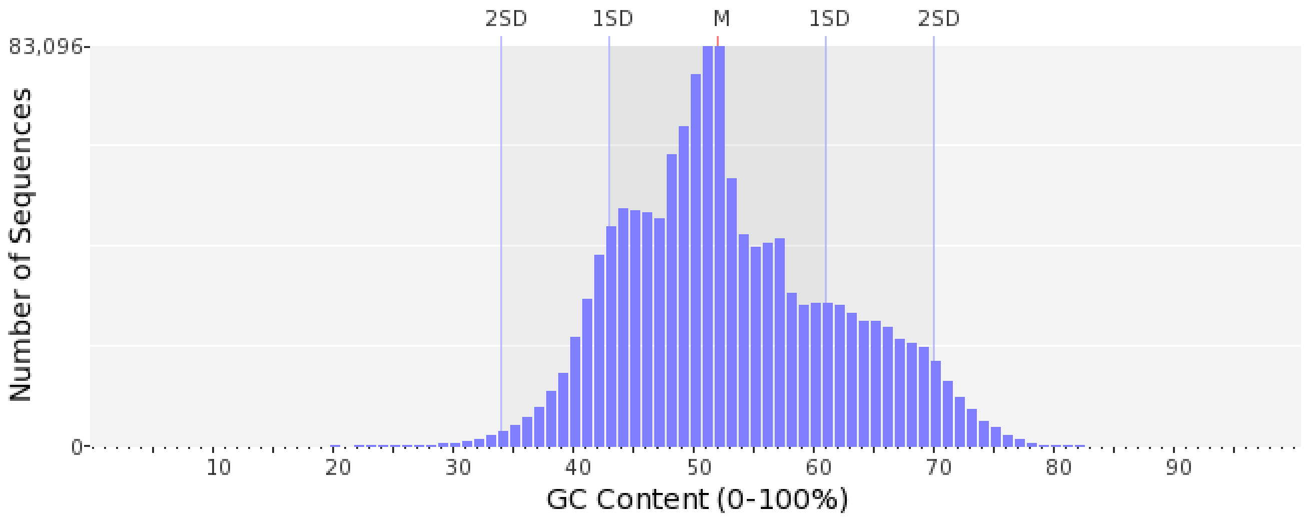

Base Quality Distribution

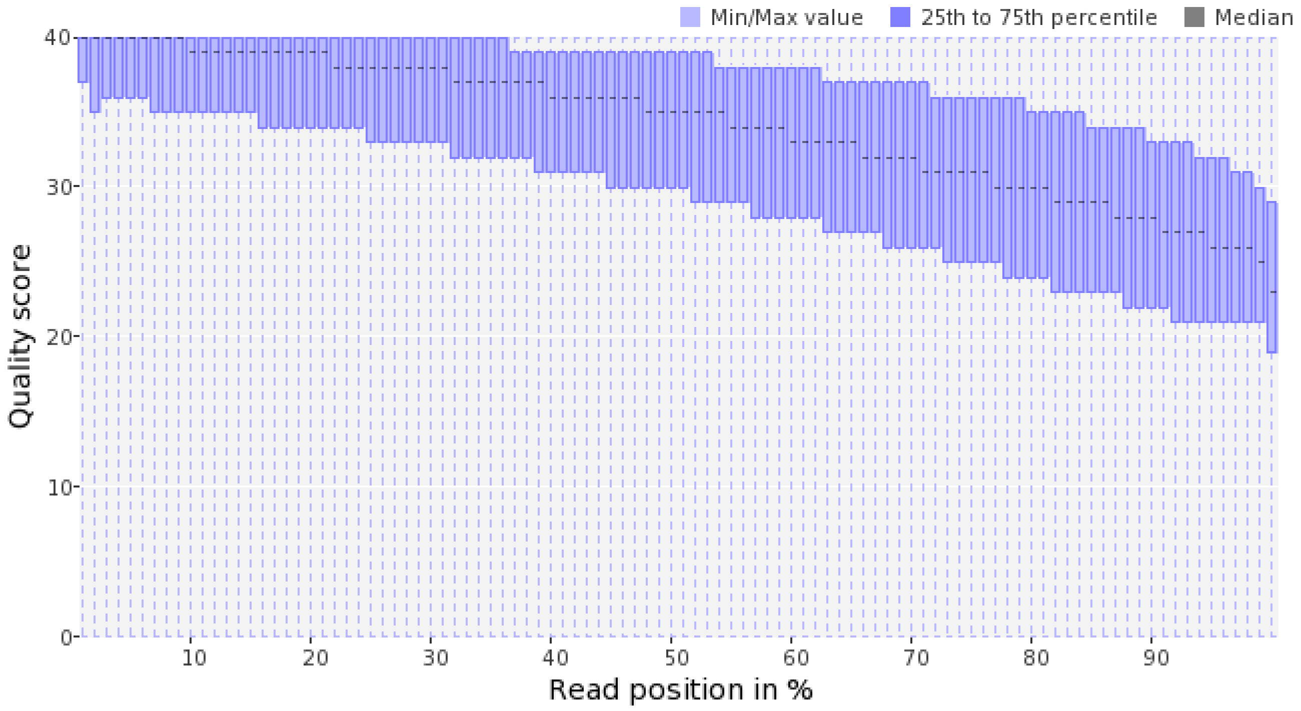

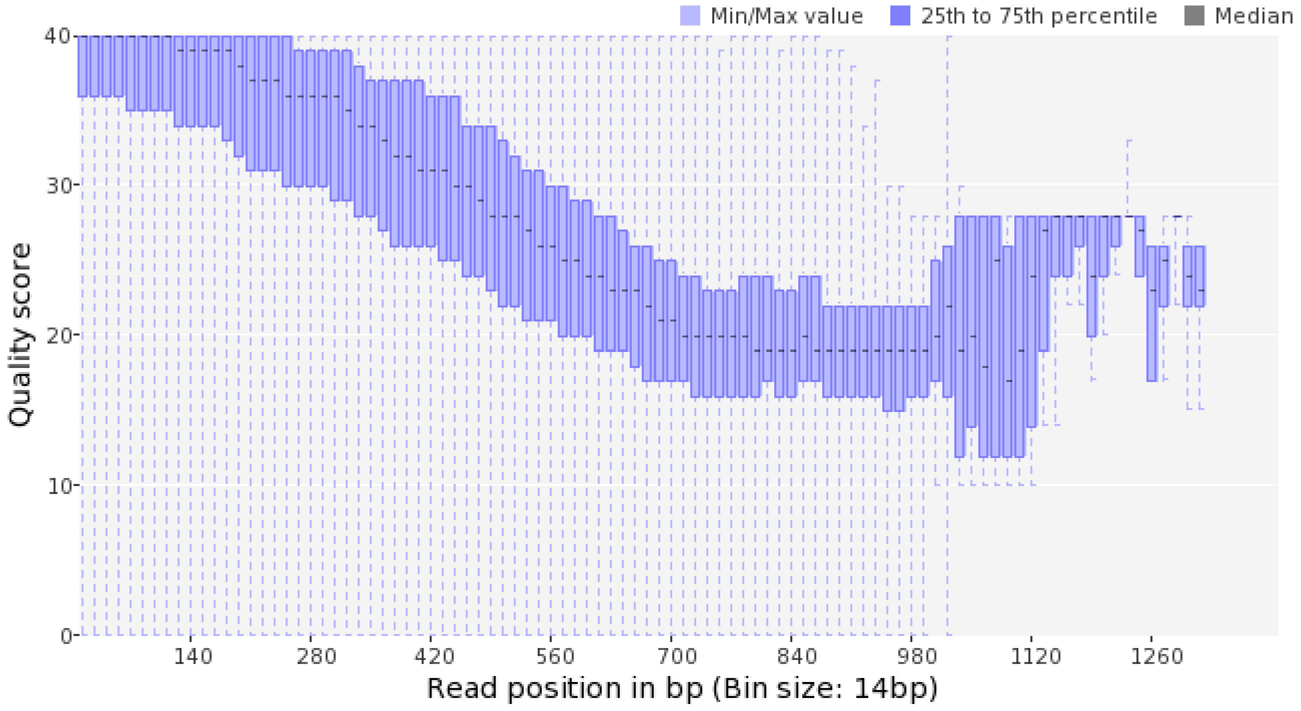

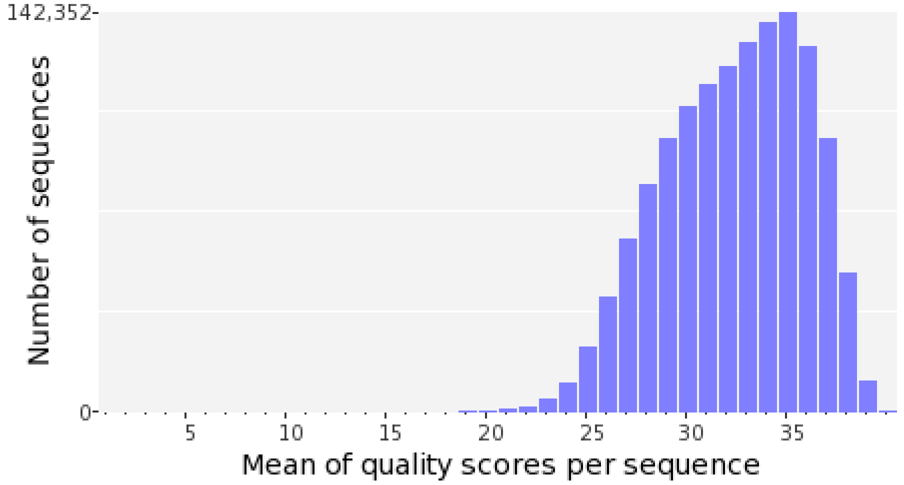

Occurence of N

Sequences with N:	**56,896 (4.13 %)**
Max percentage of Ns per sequence:	**40 %**

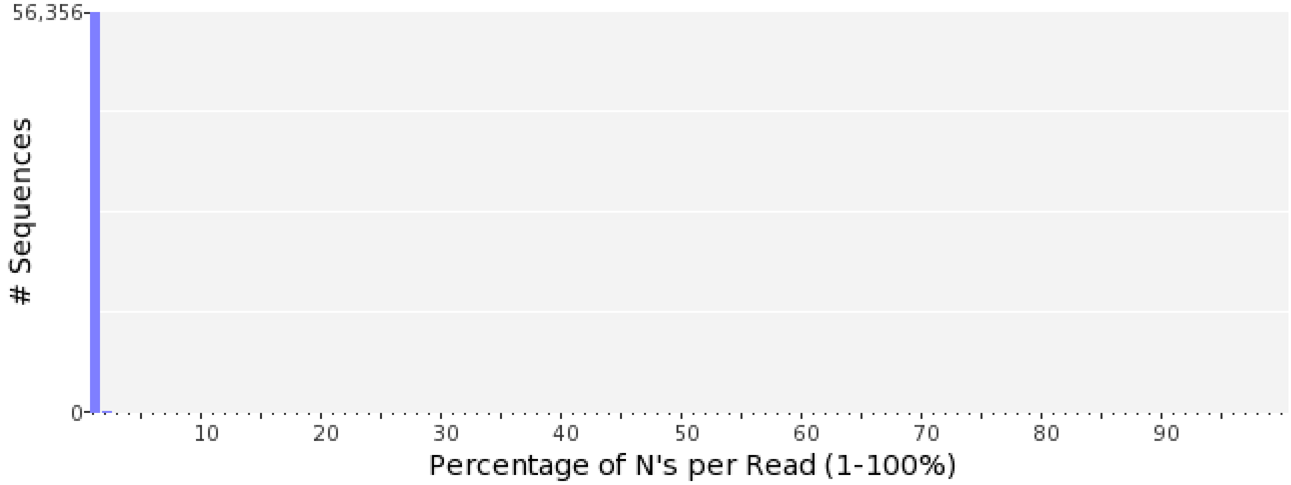

Poly-A/T Tails

	5'-end	3'-end
Sequences with tail:	**3,840 (0.28 %)**	**11,444 (0.83 %)**
Maximum tail length:	**123**	**315**

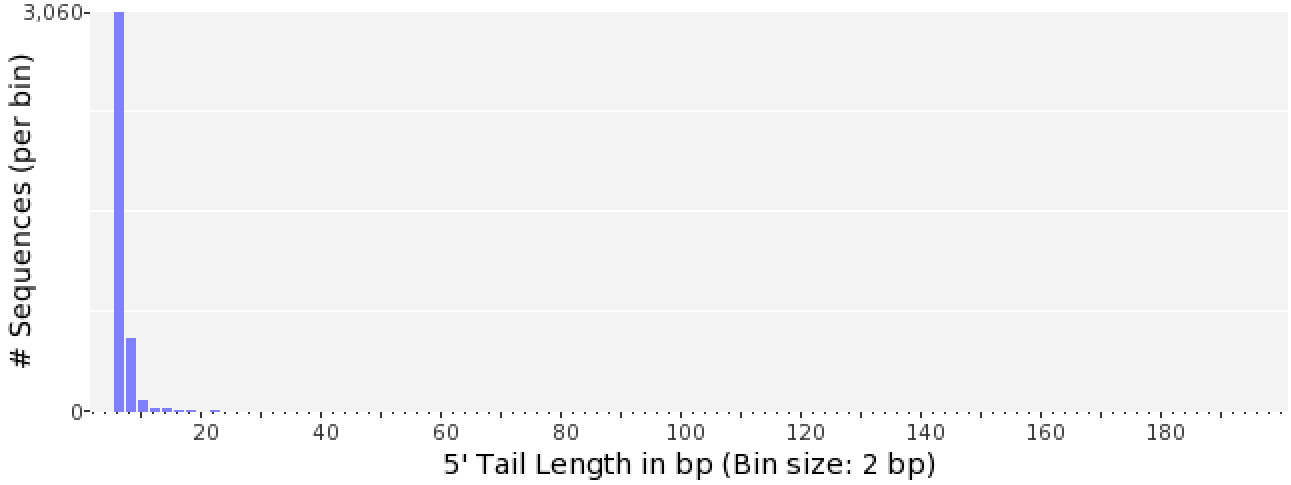

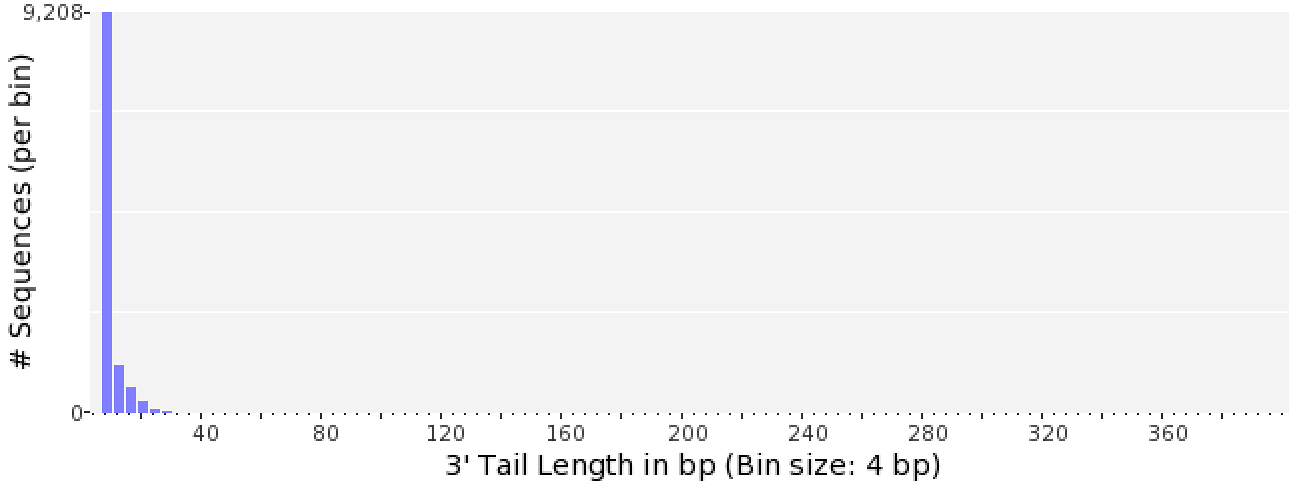

Tag Sequence Check

	5'-end	3'-end
Probability of tag sequence:	0 %	12 %
GSMIDs or RLMIDs:	none	

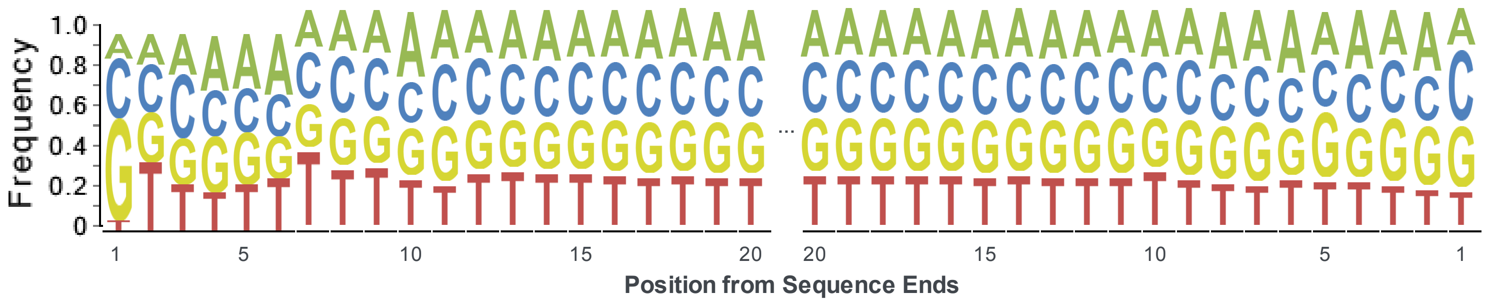

Sequence Duplication

	# Sequences	Max duplicates
Exact duplicates:	58,280 (4.23 %)	215
Exact duplicates with reverse complements:	101 (0.01 %)	1
5' duplicates	125,279 (9.09 %)	14
3' duplicates	24,129 (1.75 %)	45
5'/3' duplicates with reverse complements	12,978 (0.94 %)	3
Total:	220,767 (16.01 %)	-

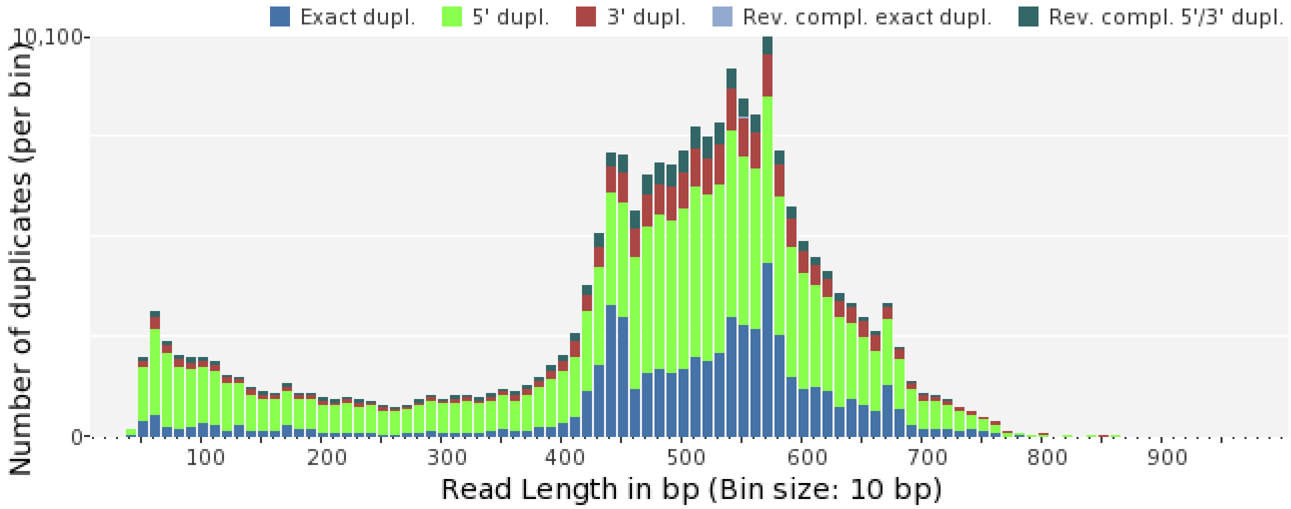

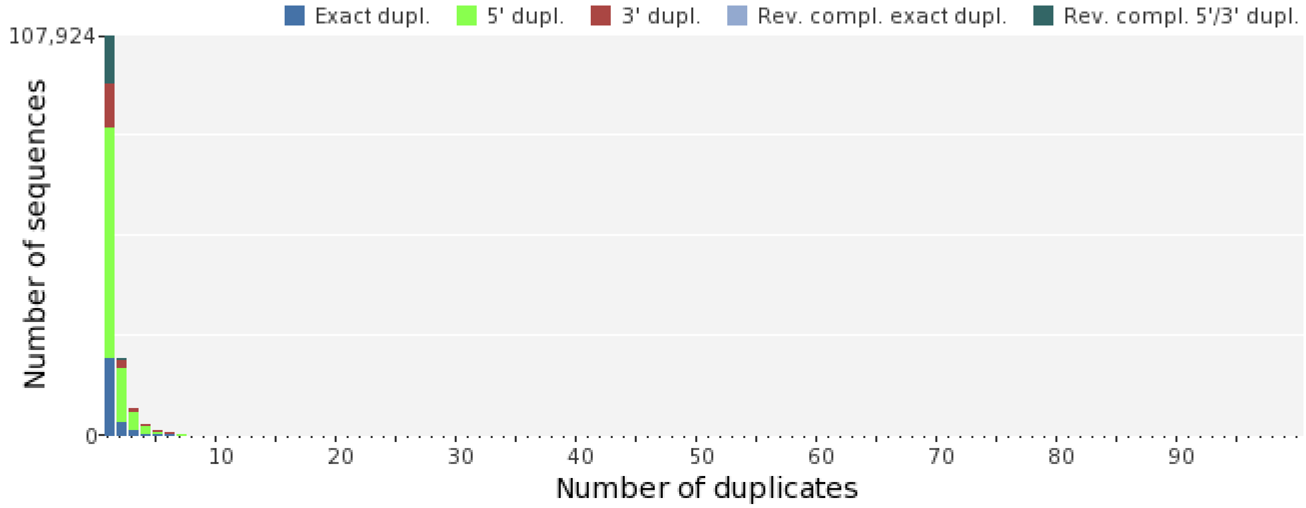

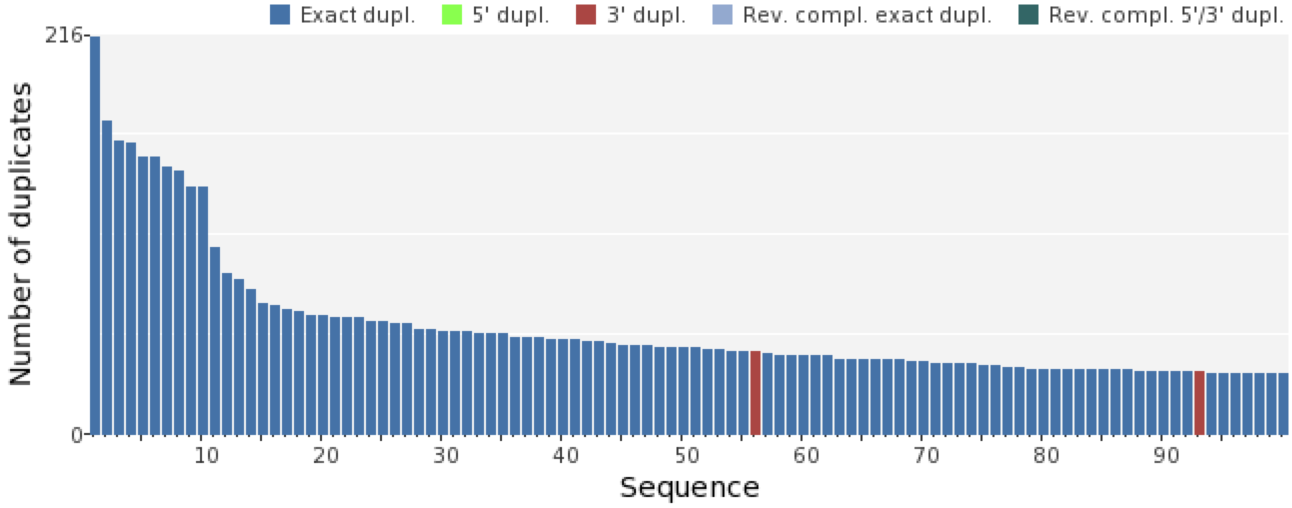

Sequence Complexity

	Value	Sequence
Minimum DUST score:	**0**	CCAACAACTGAATGGAGACACCTATACTTCTAGGACGCCATTTGC

Maximum DUST score:	**97**	ATTTTTTTTTTTTTTTTTTTTTTTTTTTTTTTTTTTTTTTTTTTTTTTTTTTTTTTTTTTTTTT TTTTTTTTT
Minimum Entropy value:	**1**	ATTTTTTTTTTTTTTTTTTTTTTTTTTTTTTTTTTTTTTTTTTTTTTTTTTTTTTTTTTTTTTT TTTTTTTTT
Maximum Entropy value:	**98**	CTTGAGACTAACCCGTGCGACACGGGTTAGTCTCAAG

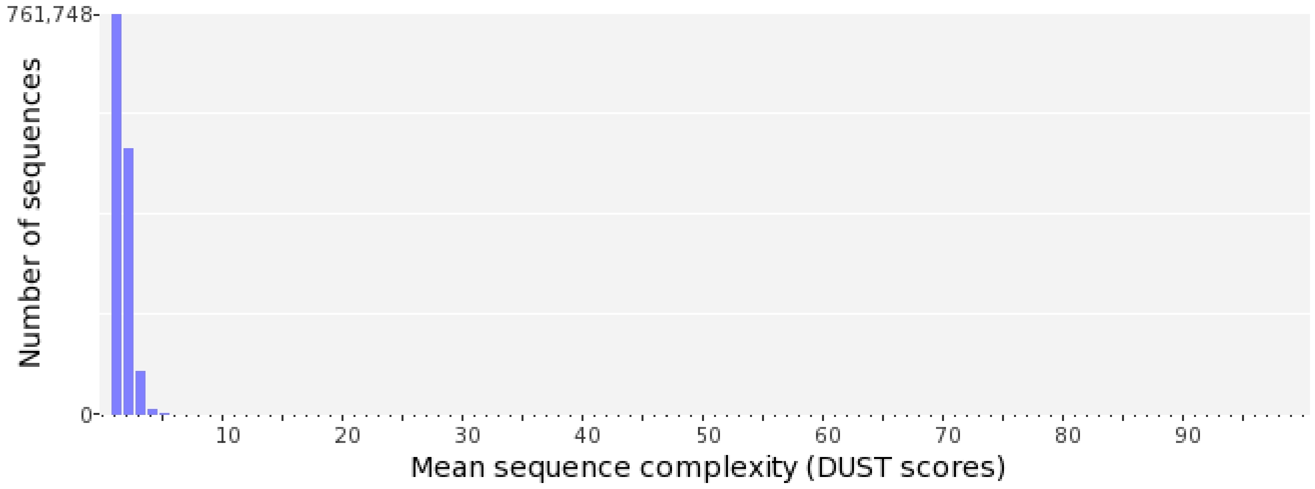

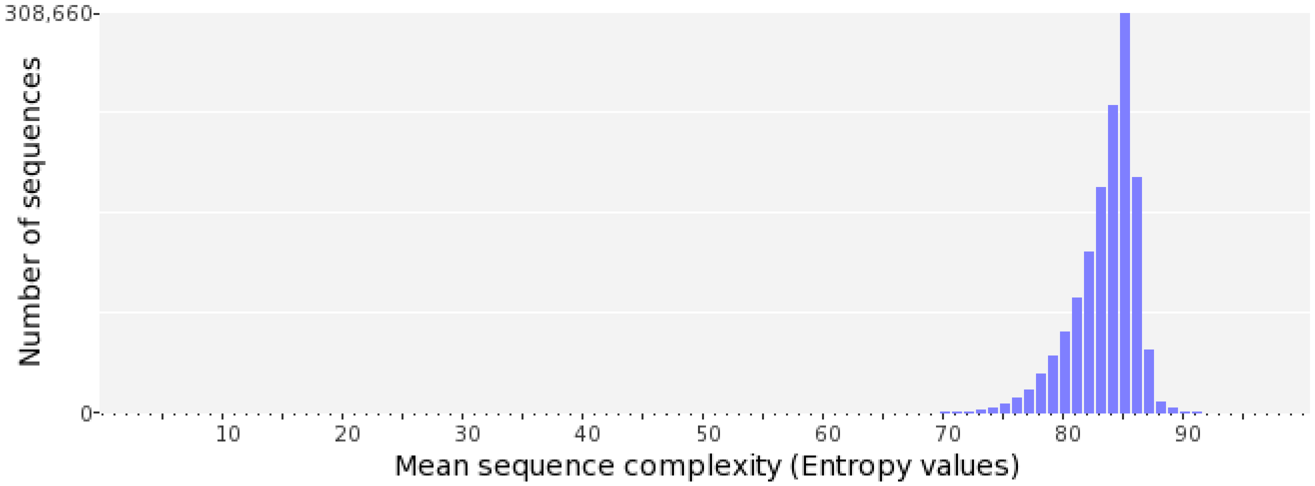

Dinucleotide Odds Ratios

	AA/TT	AC/GT	AG/CT	AT	CA/TG	CC/GG	CG	GA/TC	GC	TA
Odds ratio	**1.0604**	**0.8773**	**1.0757**	**0.9872**	**1.1338**	**0.9785**	**0.7912**	**1.0998**	**1.0623**	**0.6710**

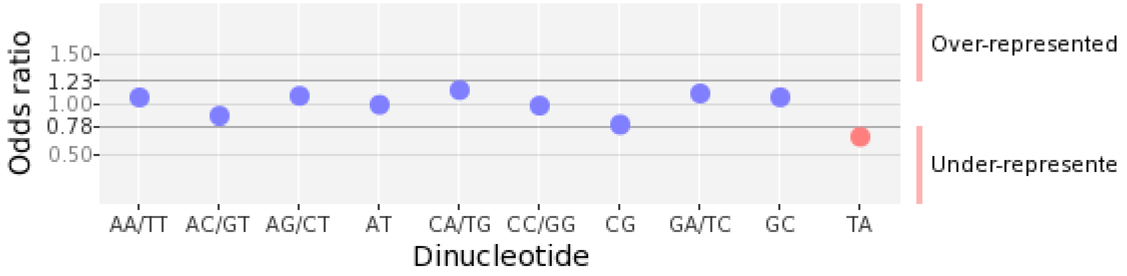

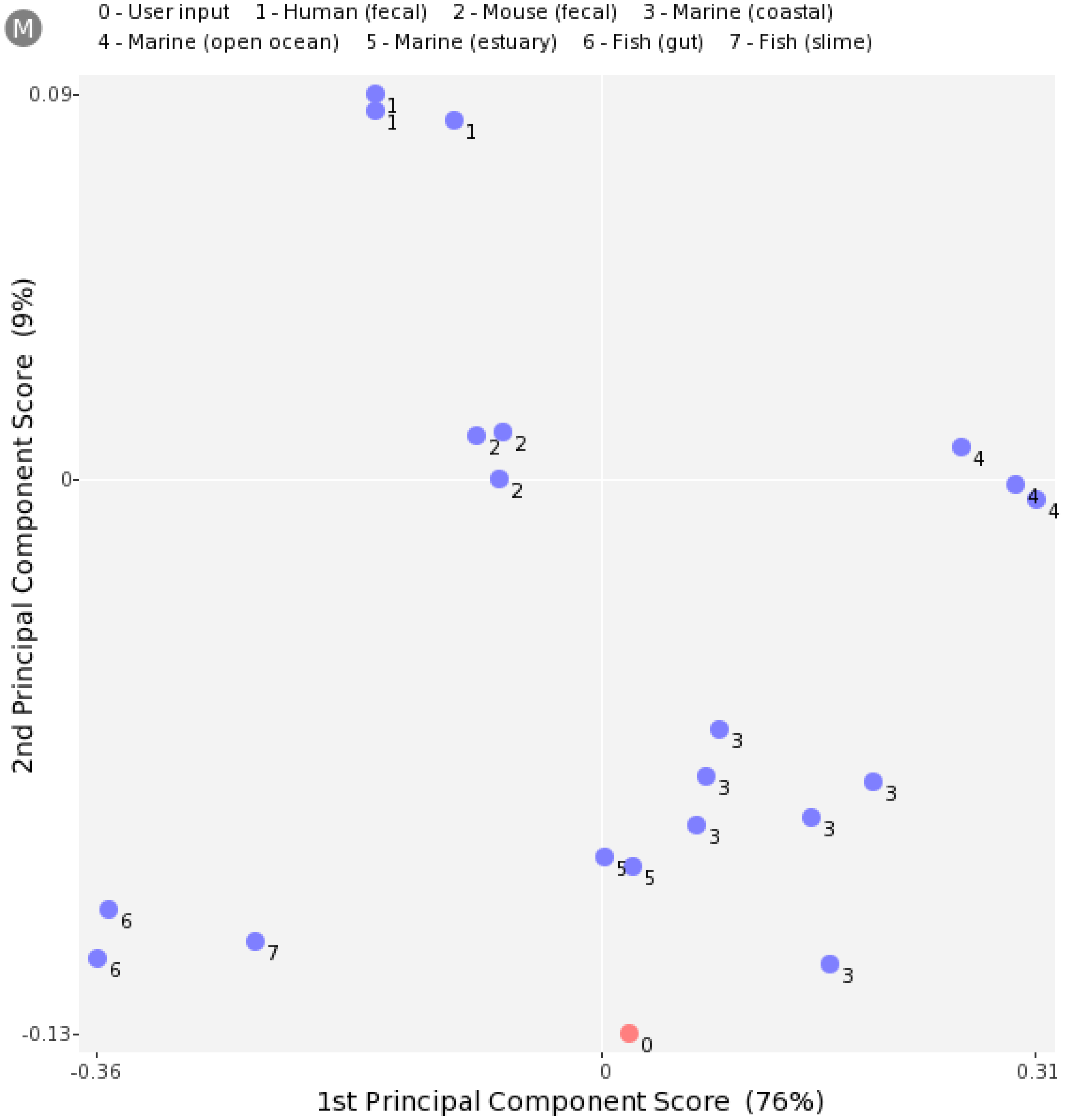
M
0 - User input
1 - Human (fecal)
2 - Mouse (fecal)
3 - Marine (coastal)
4 - Marine (open ocean)
5 - Marine (estuary)
6 - Fish (gut)
7 - Fish (slime)
2nd Principal Component Score (9%)
1st Principal Component Score (76%)
0.09
0
-0.13
-0.36
0
0.31

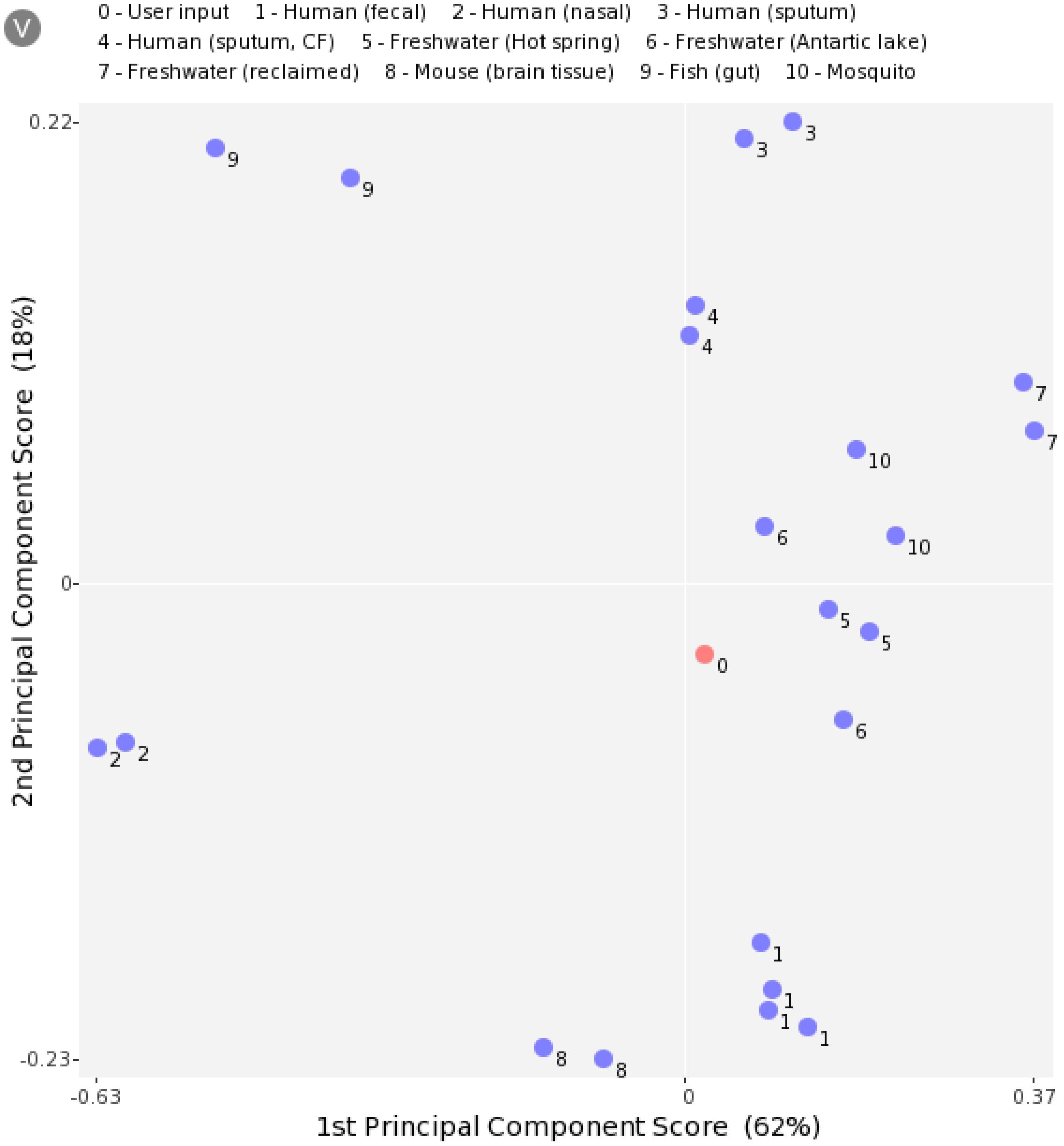
V
0 - User input
1 - Human (fecal)
2 - Human (nasal)
3 - Human (sputum)
4 - Human (sputum, CF)
5 - Freshwater (Hot spring)
6 - Freshwater (Antartic lake)
7 - Freshwater (reclaimed)
8 - Mouse (brain tissue)
9 - Fish (gut)
10 - Mosquito
0.22
0
-0.23
-0.63
0
0.37
2nd Principal Component Score (18%)
1st Principal Component Score (62%)
