# Supplementary material for: A reference floral transcriptome of sexual and apomictic Paspalum notatum
Source: BMC Genomics. 2017 Apr 21;18:318. doi: 10.1186/s12864-017-3700-z (PMC5399859; doi:10.1186/s12864-017-3700-z)
Supplement: Supplementary file 5 — BLAST analysis of cDNA sequences associated to apomixis identified by Laspina et al. (2008) against the 454/Roche global library. (DOC 37 kb) [file 12864_2017_3700_MOESM5_ESM.doc]

**Table S4: BLAST analysis of cDNA sequences associated to apomixis identified by Laspina et al. (2008) onto the 454/Roche global library**

**_____________________________________________________________________________________________________________________________________________**

**DET* ISOTIG* ISOGROUP* LENGTH* NUMCONTIGS* EXPECT* ANNOTATION***

_____________________________________________________________________________________________________________________________________________

N5 isotig32760 gene=isogroup13985 length=1622 numContigs=1 0.0 Q9ZQ34 Uncharacterized protein (similar to LUNAPARKB)

**N7** isotig00325 gene=isogroup00014 length=4386 numContigs=7 e-180 Q5RES6 Trafficking protein particle complex subunit 2

isotig00324 length=5405 numContigs=6 e-124

**N12** isotig09111 gene=isogroup00943 length=1320 numContigs=3 e-109 Q8W496 Protochlorophyllide-dependent translocon comp. 52

isotig09110 length=1363 numContigs=3 e-109

isotig09108 length=1893 numContigs=4 e-109

isotig09107 length=1936 numContigs=4 e-109

**N15** isotig13516 gene=isogroup02094 length=2730 numContigs=2 0.0 O65351 Subtilisin-like protease

isotig13515 length=2771 numContigs=3 0.0

isotig13514 length=2983 numContigs=3 0.0

**N17** isotig54694 gene=isogroup35919 length=537 numContigs=1 e-160 P31110 Thaumatin-like protein

**N18** isotig02025 gene=isogroup00072 length=1679 numContigs=4 e-112 ---

isotig02024 length=1969 numContigs=5 e-112

isotig02022 length=2804 numContigs=5 e-112

isotig02021 length=3556 numContigs=7 e-112

isotig02020 length=4548 numContigs=8 e-112

**N20** isotig37965 gene=isogroup19190 length=1105 numContigs=1 0.0 B3GS44 GPI-anchored protein LORELEI

**N26** isotig57579 gene=isogroup38804 length=487 numContigs=1 1e-09 ---

**N43** isotig02027 gene=isogroup00072 length=1231 numContigs=2 e-157 ---

isotig02026 length=1478 numContigs=3 e-153

isotig02025 length=1679 numContigs=4 e-153

isotig02024 length=1969 numContigs=5 e-153

isotig02022 length=2804 numContigs=5 e-153

isotig02021 length=3556 numContigs=7 e-153

isotig02020 length=4548 numContigs=8 e-153

**N46** isotig05824 gene=isogroup00409 length=2107 numContigs=3 0.0 ---

isotig05823 length=2183 numContigs=4 0.0

isotig05822 length=2400 numContigs=4 0.0

isotig05821 length=2417 numContigs=4 0.0

isotig05820 length=2411 numContigs=4 0.0

isotig05819 length=2476 numContigs=5 0.0

isotig05818 length=2493 numContigs=5 0.0

isotig05817 length=2487 numContigs=5 0.0

**N51** isotig21661 gene=isogroup05766 length=1005 numContigs=2 1e-70 COG5066 VAMP-assoc. protein inv. in inositol metabolism

isotig21660 length=1865 numContigs=2 1e-70

**N54** isotig39511 gene=isogroup20736 length=1000 numContigs=1 e-130 P0CH33 Polyubiquitin 11

**N56** isotig34289 gene=isogroup15514 length=1427 numContigs=1 e-115 ---

**N58** isotig13529 gene=isogroup02099 length=4323 numContigs=2 4e-92 PF04783.7 DUF630 Protein of unknown function (DUF630)

**N60** isotig28268 gene=isogroup09493 length=3107 numContigs=1 0.0 Q29AK2 Leishmanolysin-like peptidase

**N69** isotig41533 gene=isogroup22758 length=896 numContigs=1 0.0 P85107 Trimethylguanosine synthase

**N95** isotig31796 gene=isogroup13021 length=1766 numContigs=1 1e-42 P46280 Elongation factor Tu

**N98** isotig58251 gene=isogroup39476 length=471 numContigs=1 6e-17 Q86U02 Putative uncharacterized protein

**N99** isotig01876 gene=isogroup00064 length=7131 numContigs=7 2e-91 P04146 Copia protein

isotig01875 length=7135 numContigs=8 2e-91

isotig01872 length=8306 numContigs=9 2e-91

isotig01871 length=8310 numContigs=10 2e-91

isotig01870 length=8692 numContigs=8 2e-91

isotig01869 length=8696 numContigs=9 2e-91

**N108** isotig29883 gene=isogroup11108 length=2193 numContigs=1 0.0 Q9SZL8 Protein FAR1-RELATED SEQUENCE 5

**N114** isotig28413 gene=isogroup09638 length=2962 numContigs=1 e-141 O81635 Kinesin-4

**N115** isotig22060 gene=isogroup05966 length=1989 numContigs=2 2e-24 Q96EQ0 Small glutamine-rich TTP repeat-cont. protein

**N116** isotig29523 gene=isogroup10748 length=2324 numContigs=1 7e-95 Q9NQG5 Reg. of nuclear pre-mRNA domain-cont. protein 1B

**N119** isotig30535 gene=isogroup11760 length=1997 numContigs=1 1e-53 Q84KG5 Carotenoid 9,10(9',10')-cleavage dioxygenase

*DET: Differentially Expressed Tag. ISOTIG: allele/splice variant. ISOGROUP: gene. LENGTH: length of the transcript in the 454 database. CONTIGS: number of contigs that integrates the isotig. Expect: E-value of the BLASTN search onto the ALL isotigs database. ANNOTATION: top BLASTX in databases.
