# Supplementary material for: A reference floral transcriptome of sexual and apomictic Paspalum notatum
Source: BMC Genomics. 2017 Apr 21;18:318. doi: 10.1186/s12864-017-3700-z (PMC5399859; doi:10.1186/s12864-017-3700-z)
Supplement: Supplementary file 8 — Protein-protein interaction clusters affected during the transition from sexuality to apomixis. (DOC 37 kb) [file 12864_2017_3700_MOESM8_ESM.doc]

Protein-protein interaction clusters affected during the transition from sexuality to apomixis

| **Cluster*** | **Genes** | **Name** |
| --- | --- | --- |
| 1 | AT4G19030  AT3G16310  AT4G35580  AT1G79040  AT5G08080  AT3G50910  AT5G22290  AT1G27300  AT3G44220  AT3G47040  AT4G38970  AT5G22880  AT2G36460  AT2G18960  AT2G39050  AT2G46700  AT1G02950  AT2G24520  AT2G36190  AT1G64200  AT3G15800  AT2G17660  AT5G56010  AT3G52600  AT3G59790  AT2G01760  AT3G49160  AT4G19760  AT2G47710  AT3G55120  AT3G51240  AT5G13930  AT3G11930  AT2G44970 | NOD26-LIKE MAJOR INTRINSIC PROTEIN 1  MITOTIC PHOSPHOPROTEIN N' END (MPPN) FAMILY PROTEIN  CALMODULIN-BINDING NAC PROTEIN  PHOTOSYSTEM II SUBUNIT R, PSBR  ATSYP132, SYNTAXIN OF PLANTS 132, SYP132  UNKNOWN PROTEIN  ANAC089, FRUCTOSE-SENSING QUANTITATIVE TRAIT LOCUS 6  UNKNOWN PROTEIN  LEA HYDROXYPROLINE-RICH GLYCOPROTEIN FAMILY  GLYCOSYL HYDROLASE FAMILY PROTEIN  ATFBA2, FBA2, FRUCTOSE-BISPHOSPHATE ALDOLASE 2  H2B, HISTONE B2, HISTONE H2B, HTB2  FBA6, FRUCTOSE-BISPHOSPHATE ALDOLASE 6  PLASMA MEMBRANE PROTON ATPASE, PMA  ARATHEULS3, EULS3, EUONYMUS LECTIN S3  ATCRK3, CDPK-RELATED KINASE 3, CRK3  ATGSTF4, GLUTATHIONE S-TRANSFERASE 31  AHA5, H(+)-ATPASE 5, HA5  ATCWIN4, ATCWINV4, CELL WALL INVERTASE 4, CWINV4  VACUOLAR H+-ATPASE SUBUNIT E ISOFORM 3, VHA-E3  GLYCOSYL HYDROLASE SUPERFAMILY PROTEIN  RPM1-INTERACTING PROTEIN 4 (RIN4) FAMILY PROTEIN  HEAT SHOCK PROTEIN 81-3  ATCWIN2, ATCWINV2, CELL WALL INVERTASE 2, CWINV2  ATMPK10, MAP KINASE 10, MPK10  ARR14, RESPONSE REGULATOR 14, RR14  UNKNOWN PROTEIN  GLYCOSYL HYDROLASE FAMILY PROTEIN  ADENINE NUCLEOTIDE ALPHA HYDROLASES-LIKE PROTEIN  CHALCONE FLAVANONE ISOMERASE  FLAVANONE 3-HYDROXYLASE  CHALCONE SYNTHASE, CHS  ADENINE NUCLEOTIDE ALPHA HYDROLASES-LIKE PROTEIN  ALPHA/BETA-HYDROLASES SUPERFAMILY PROTEIN |
| 2 | AT4G12800  AT2G46820  AT2G22125  AT3G54390  AT5G63450  AT5G55290  AT2G35580  AT1G55490  AT2G39730  AT3G17840  AT5G19140  AT5G50260  AT3G45640 | PHOTOSYSTEM I SUBUNIT L, PSAL  CURVATURE THYLAKOID 1B, PHOTOSYSTEM I P SUBUNIT  CELLULOSE SYNTHASE-INTERACTIVE PROTEIN 1  SEQUENCE-SPECIFIC DNA BINDING TRANSCRIPTION FACTOR  CYTOCHROME P450, FAMILY 94, SUBFAMILY B  ATPASE, V0 COMPLEX, SUBUNIT E  SERINE PROTEASE INHIBITOR (SERPIN) FAMILY PROTEIN  CHAPERONIN 60 BETA  RCA, RUBISCO ACTIVASE  RECEPTOR-LIKE KINASE 902, RLK902  AILP1, ATAILP1  CEP1, CYSTEINE ENDOPEPTIDASE 1  MITOGEN-ACTIVATED PROTEIN KINASE 3, MPK3 |
| 3 | AT3G27960  AT5G20250  AT4G29820  AT1G55740  AT5G03650  AT5G44120  AT2G43710 | KINESIN LIGHT CHAIN-RELATED 2, KLCR2  DARK INDUCIBLE 10, DIN10, RAFFINOSE SYNTHASE 6, RS6  HOMOLOG OF CFI-25, A POLYADENYLATION FACTOR SUBUNIT  ATSIP1, RAFFINOSE SYNTHASE 1, RS1, SEED IMBIBITION 1, SIP1  SBE2.2, STARCH BRANCHING ENZYME 2.2  ATCRA1, CRA1, CRU1, CRUCIFERINA  FATTY ACID BIOSYNTHESIS 2, STEAROYL-ACP DESATURASE |
| 4 | AT4G18730  AT1G78270  AT4G18430 | RIBOSOMAL PROTEIN L16B, RPL16B  ATUGT85A4, UDP-GLUCOSYL TRANSFERASE 85A4, UGT85A4  ATRABA1E, RAB GTPASE HOMOLOG A1E, RABA1E |
| 5 | AT4G14570  AT1G34220 | AARE, ACYLAMINO ACID-RELEASING ENZYME, ATAARE  IST1-LIKE 1, ISTL1 |

*Occurrence of protein-protein interaction networks were investigated in the *Arabidopsis* putative orthologs of the 200 top ranked (based on FDR) differentially expressed candidates.
